# Endothelial Senescence-Associated Secretory Signaling Promotes Macrophage Extracellular Traps Formation and Contributes to the Exacerbation of Combined Lung Injury

**DOI:** 10.7150/ijbs.133943

**Published:** 2026-07-20

**Authors:** Wentao Xie, Hongsen Liao, Yali Dai, Lin Chen, Yujie Li, Zhuo Cheng, Chunmeng Shi

**Affiliations:** Institute of Rocket Force Medicine, State Key Laboratory of Trauma and Chemical Poisoning, Army Medical University, Chongqing 400038, China

**Keywords:** radiation-induced lung injury, macrophage extracellular traps, endothelial senescence, senescence-associated secretory signaling, pulmonary fibrosis, Cordycepin

## Abstract

Radiation-induced lung injury (RILI) is a common complication of thoracic radiotherapy and can be critically exacerbated by pre-existing pulmonary inflammation, yet the synergistic mechanisms driving this pathology remain elusive. Using a murine model of combined lung injury induced by lipopolysaccharide (LPS) and thoracic irradiation (IR), we identified macrophage extracellular traps (METs), a type of web-like chromatin structure released by macrophages, rather than neutrophil extracellular traps (NETs), as a prominent pathogenic process. Mechanistically, the combination of LPS and irradiation induced an early endothelial senescence-associated phenotype and a CXC chemokine-enriched secretory profile. These signals engaged macrophage CXCR2, leading to p38/ERK pathway activation and reactive oxygen species (ROS) production that contributed to METs formation (METosis). Functionally, METs serve as potential dual-phase mediators, contributing to epithelial barrier disruption during acute injury and promoting epithelial-mesenchymal transition (EMT)-like epithelial remodeling, thereby potentially linking early inflammatory damage to subsequent fibrotic progression. Furthermore, we demonstrate that the bioactive compound Cordycepin exerts protective effects by suppressing p38/ERK pathway activation and attenuating METosis. Collectively, these findings support a potential endothelial senescence-associated secretory signaling-METosis axis, providing a novel mechanistic framework and candidate therapeutic strategies for managing high-risk radiation-induced lung injury.

## Introduction

Thoracic radiotherapy is a common treatment for multiple malignancies, including lung, breast, and esophageal cancers, but its clinical application is often impeded by radiation-induced lung injury (RILI), a dose-limiting toxicity 1. Clinical data indicate that approximately 5-25% of patients undergoing thoracic radiotherapy will develop RILI 2-4, which presents with a characteristic biphasic pathology: an initial phase of radiation pneumonitis (RP), dominated by inflammatory infiltration, followed by a potential progression to pulmonary fibrosis (PF) 2, a typically irreversible condition characterized by tissue remodeling and scar formation.

The risk and severity of RILI are influenced by multiple factors and exhibit significant inter-individual variability 5. In clinical practice, patients with thoracic malignancies may present with infection or a persistent infection-related inflammatory state before or during radiotherapy. Emerging clinical evidence suggests that such a baseline inflammatory background is closely associated with an increased risk of subsequent radiation-induced lung injury and adverse clinical outcomes. Previous studies have shown that elevated pre-radiotherapy plasma IL-6 levels predict a higher risk of radiation pneumonitis 6, while increased pre-treatment CRP is associated with severe radiation pneumonitis 7. Consistently, increased [18F]FDG uptake in normal lung tissue before radiotherapy, reflecting pre-existing pulmonary inflammatory activity, has also been linked to subsequent radiation pneumonitis 8. Together, these observations suggest that a pre-existing pulmonary inflammatory state can be an important determinant of the risk and severity of lung injury after thoracic irradiation. However, the precise mechanisms driving this synergistic pathology remain poorly understood. Moreover, current research investigating the synergistic mechanisms between lipopolysaccharide (LPS)-induced inflammation and thoracic radiation has yielded inconsistent or conflicting conclusions 9,10, largely attributable to variations in experimental systems, dosing, and endpoints. Therefore, to address this gap, we established a murine model of severe combined lung injury by administering 2 mg/kg LPS prior to whole-thorax irradiation, a strategy designed to approximate thoracic irradiation-induced lung injury under a pre-existing inflammatory state.

Our findings show that LPS pretreatment not only significantly exacerbated early-stage radiation pneumonitis but also accelerated the development of late-stage pulmonary fibrosis. Concurrently, analysis of the immune landscape of early-stage combined lung injury revealed that macrophages, rather than neutrophils, were the predominant infiltrating cells. Given their predominance, we focused on elucidating the role of macrophages in early-stage synergistic damage. We identified macrophage extracellular traps (METs), a type of extracellular chromatin structure, as a potential pathological factor in this process. METs, functionally similar to neutrophil extracellular traps (NETs), are web-like chromatin structures released by macrophages in response to specific stimuli 11,12. While NETs are well-established drivers of acute lung injury (ALI) 13,14 and pulmonary fibrosis 15,16, the role of METs in these pathologies remains underexplored. Furthermore, we identified an early endothelial senescence-associated phenotype induced by the combined exposure to LPS and irradiation as a potential upstream event associated with METs formation (METosis).

Unlike classical replicative senescence associated with aging, this stress-induced senescence state was accompanied by a potent senescence-associated secretory phenotype (SASP) that modulates the local microenvironment. Specifically, endothelial cells exhibiting senescence-associated alterations released senescence-associated secretory chemokines that promoted macrophage activation and METosis. Functionally, METs contribute to the disruption of the epithelial barrier and promote epithelial-mesenchymal transition (EMT) remodeling, thereby potentially linking acute injury to subsequent fibrotic progression. Together, these findings support a potential endothelial senescence-associated secretory signaling-METosis axis that links pre-existing inflammation to the exacerbation of RILI. Importantly, we further show that the bioactive compound Cordycepin attenuates this pathogenic process, underscoring its potential as a therapeutic agent for combined lung injury.

## Materials and Methods

**Animal Model and Sample Collection:** Male C57BL/6 mice (20-25 g) were purchased from the Experimental Animal Center of Army Medical University. Male PAD4-knockout (PAD4⁻/⁻) mice (20-25 g) were purchased from Shulaibao Biotechnology (Wuhan, China). All animals were housed under specific pathogen-free conditions with controlled environmental settings (22 ± 2 °C, 55 ± 5% humidity, 12 h light/dark cycle) and provided with standard diet and water ad libitum. A one-week acclimatization period was allowed prior to experimental procedures. All animal protocols were approved by the Animal Research Committee of Army Medical University (approval no. AMEWEC20230024) and were conducted in accordance with institutional guidelines.

Systemic inflammation was induced via tail vein injection of lipopolysaccharide (LPS; Sigma, L2880) at 2 mg/kg, 5 mg/kg, or 10 mg/kg. For thoracic irradiation (IR), anesthetized mice (4% isoflurane) were shielded with 2-cm lead plates to selectively expose the thorax. A single 17 Gy dose was delivered at 1.3 Gy/min using a Precision X-ray irradiator. The combined injury group received thoracic irradiation 6 h after LPS injection, with the modeling strategy referencing previous studies on combined injury 10. For pharmacological interventions, Cl-amidine (10 mg/kg) (TargetMol, T10831L) was administered intraperitoneally at the time of modeling. Cordycepin (MedChemExpress, HY-N0262) was administered according to three regimens. For the acute prophylactic regimen, Cordycepin was injected intraperitoneally at 10 mg/kg once daily for 7 days before model establishment. For the acute single-dose therapeutic regimen, Cordycepin was administered intraperitoneally at 10 mg/kg at the time of model establishment. For the chronic regimen, preventive administration was performed at 10 mg/kg once daily for 7 days before model establishment, followed by therapeutic administration at 10 mg/kg every 2 days for one month after modeling. Control groups received an equivalent volume of saline.

**Histology and Lung Injury Scoring:** Lungs were fixed in 4% paraformaldehyde, dehydrated, and embedded in paraffin. Sections (4 µm) were cut using a HistoCore AUTOCUT microtome (Leica), deparaffinized, and stained with hematoxylin and eosin (H&E). Stained sections were dehydrated, cleared, and mounted. Masson's trichrome staining was performed using a modified Masson's Trichrome Staining Kit (Solarbio, G1346) according to the manufacturer's protocol. Images were captured using an Olympus VS200 microscope. For analysis, five non-overlapping fields of view were randomly selected from three mice per group. Lung injury scoring was performed following the American Thoracic Society's guidelines 17.

**Pulmonary Function Tests:** Following chamber calibration, mice were placed in restraint chambers connected to a DSI Buxco Inhalation Tower, and respiratory parameters were recorded for a 10 min period.

**Micro-Computed Tomography Scanning:** Anesthetized mice (4% isoflurane) were immobilized and scanned using a BRUKER SkyScan 1276 micro-CT scanner (BRUKER) for lung imaging.

**Evaluation of Lung Vascular Permeability:** Following intravenous injection of 70 kDa FITC-dextran (Aladdin, F491425), mice were euthanized after 2 h. Resected lungs were imaged *ex vivo* with a VISQUE InVivo Smart-LF instrument (VISQUE).

**Quantification of Protein in Bronchoalveolar Lavage Fluid (BALF):** Following euthanasia and tracheal exposure, a cannula was inserted and secured. Lungs were lavaged five times with 2 mL of ice-cold PBS (pH 7.4), with a 30s dwell time for each cycle. Collected BALF was centrifuged (300 × g, 5 min, 4 °C), and the supernatant was analyzed for protein concentration to assess alveolar leakage.

**Quantitative Real-Time Polymerase Chain Reaction (qRT-PCR):** Total RNA was extracted from cells or tissues using TRIzol reagent (Invitrogen, 15596026) and reverse-transcribed into cDNA with a reverse transcription kit (ThermoFisher, K1622). qRT-PCR was performed on a Bio-Rad CFX96 system using SYBR Green Master Mix (Takara, RR820A). Relative mRNA expression was calculated using the comparative 2^-ΔΔCt method and normalized to β-actin (ACTB). Primer sequences are listed in Table S1.

**Western Blot:** Total protein was extracted from lung tissues or cells using RIPA buffer (Beyotime, P0013B) supplemented with protease and phosphatase inhibitors (MCE, HY-K0010, HY-K0023). After protein quantification (BCA assay), equal amounts of protein were separated by SDS-PAGE on 4-20% precast gels (GenScript, M00657) and transferred to PVDF membranes. Membranes were blocked, incubated with primary antibodies (overnight, 4 °C), and then with secondary antibodies (1 h, RT). Bands were visualized with ECL reagent (Merck Millipore, WBKLS0500) on a ChemiDoc Imaging System (Bio-Rad). Membranes were stripped (EpiZyme, PS107S) and reprobed for β-Actin. Band intensities were quantified using ImageJ. Antibodies are listed in Table S2.

**Enzyme-Linked Immunosorbent Assay (ELISA):** Serum samples and culture supernatants were diluted at a ratio of 1:10. The assays were performed using commercial ELISA kits specific for mouse TNF-α (Elabscience, E-EL-M0049), mouse IL-6 (Elabscience, E-EL-M0044), and mouse MPO-DNA (MEIMIAN, MM-1177M2) according to the manufacturers' protocols.

**Immunofluorescence (IF):** Single and multiplex IF were performed on paraffin-embedded lung sections or cultured cells. After deparaffinization and antigen retrieval, sections were blocked and incubated with primary antibodies (overnight, 4 °C), followed by corresponding secondary antibodies (1 h, RT). TUNEL staining was performed using an *in situ* cell death detection kit according to the manufacturer's protocol (Roche, 11684817910, Switzerland). Nuclei were counterstained with DAPI (Beyotime, C1006). Images were acquired with an FV300 confocal microscope and analyzed using ImageJ for positive cell counts or mean fluorescence intensity (MFI). Antibodies are listed in Table S3.

**Flow Cytometry Analysis:** Single-cell suspensions were prepared from lung tissue using a Lung Dissociation Kit (Miltenyi, 130-095-927). Cells were treated with an Fc receptor blocking reagent (Biolegend, 156604), stained with Zombie Aqua live/dead dye (Biolegend, 423101), and then stained for surface markers. For intracellular targets, cells were fixed and permeabilized using a Foxp3/Transcription Factor Staining Buffer Set (Invitrogen, 00-5523-00) before antibody staining. Data were acquired on an Attune NxT cytometer (Invitrogen) and analyzed with FlowJo software. Antibodies are listed in Table S4.

**RNA Sequencing Data Analysis:** One day post-modeling, lung tissues from control, IR, LPS, and LPS+IR groups were collected for RNA sequencing (Seqhealth Technology, Wuhan). After data processing, differentially expressed genes (DEGs) analysis was conducted in R (v4.3.3) using limma, with significance defined as |log2FC| > 1 and p.adj < 0.05. Subsequent functional analyses and visualizations included GO (Gene Ontology) and Kyoto Encyclopedia of Genes and Genomes (KEGG) enrichment (ClusterProfiler), Gene Set Enrichment Analysis (GSEA) (GseaVis), volcano plots (ggplot2), and heatmaps (pheatmap). All analyses were conducted in R (v4.3.3). In addition, endothelial cells from the control, IR, LPS, and LPS+IR groups were collected at 24 h for RNA sequencing by Seqhealth Technology, Wuhan. DEGs analysis of endothelial cells was performed using limma in R (v4.3.3), with significance defined as |log₂FC| > 3 and adjusted p < 0.01. The commonly upregulated genes from these three comparisons were then intersected with the SenMayo SASP gene set 18.

**Near-Infrared Fluorescence Imaging of Pulmonary Fibrosis with IR-780:** Mice were injected i.p. with IR-780 (0.04 mg/mL; Sigma-Aldrich, 425311) 48 h before sacrifice. Excised lungs were imaged *ex vivo* using a VISQUE InVivo Smart-LF system. For histology, sections were counterstained with a nuclear dye, imaged with an FV300 confocal microscope, and the IR-780-positive area was quantified using ImageJ.

**Cell Culture and Co-culture Experiment:** Primary human umbilical vein endothelial cells (HUVEC), primary mouse pulmonary microvascular endothelial cells (MPVEC), the human monocytic THP-1 cell line, and the human lung adenocarcinoma A549 cell line were all purchased from Procell. HUVEC and MPVEC were cultured in Endothelial Cell Medium (ECM). A549 cells were cultured in F-12K medium. THP-1 cells were cultured in RPMI-1640 medium supplemented with 0.05 mM β-mercaptoethanol. Differentiation into macrophage-like cells was induced by treatment with 20 nM phorbol 12-myristate 13-acetate (PMA) for 48 h. All cells were maintained at 37 °C in a humidified atmosphere of 5% CO₂.

**Endothelial-Macrophage Co-culture via Conditioned Medium:** Endothelial cells (HUVEC or MPVEC) were divided into four groups: Control, IR (6 Gy), LPS (1 µg/mL), and LPS+IR. After 24 h of stimulation, the medium was replaced with fresh medium for an additional 24 h. The collected supernatants were concentrated by ultracentrifugation and then added to differentiated THP-1 macrophages or BMDMs for overnight incubation.

**Macrophage-Epithelial Co-culture via Conditioned Medium:** Differentiated THP-1 macrophages were stimulated overnight with 100 ng/ml recombinant CXCL8 to induce the formation of METs. The resulting METs-containing supernatant was collected and added to A549 cells.

**Fluorescence Staining and Imaging of METs:** For visualization, cells were co-stained with the cell-permeable nuclear dye Hoechst 33342 and the cell-impermeable extracellular DNA dye SYTOX Green. Images were acquired using an Olympus FV300 confocal microscope.

**Inhibition of METs Formation:** To investigate the signaling pathways involved in METs formation, macrophages were treated with anti-CXCL8 neutralizing antibody, anti-Pan-GRO neutralizing antibody, CXCR2 inhibitors (SB225002 and Danirixin), p38 MAPK inhibitor (SB202190), ERK inhibitor (PD98059), PI3K inhibitor (LY294002), NADPH oxidase inhibitor (DPI). The effect of each treatment on METs formation was subsequently assessed to determine the role of the targeted molecule.

**CRISPR-Cas9-Mediated CXCR2 Gene Knockdown:** A CXCR2-knockdown THP-1 cell line was generated using a lentiviral CRISPR-Cas9 system. An sgRNA targeting a human CXCR2 exon was cloned into a lentiviral vector co-expressing Cas9 and GFP. Lentivirus was produced in HEK293T cells and used to infect THP-1 cells. Transduced (GFP-positive) cells were enriched by fluorescence-activated cell sorting (FACS). Knockdown efficiency was confirmed by genomic DNA sequencing and at the protein level by Western blot.

**Statistical Analysis:** Statistical analyses were performed using GraphPad Prism 10.6.1. Data are presented as mean ± standard deviation (SD). For comparisons between two groups, an unpaired two-tailed Student's t-test was used. For comparisons among three or more groups, one-way analysis of variance (ANOVA) was performed, followed by Dunnett's multiple-comparisons test. Statistical significance was defined as P < 0.05 (ns, not significant; *P < 0.05; **P < 0.01; ***P < 0.001).

## Results

### Combination of LPS and Thoracic Irradiation Exacerbates Acute Lung Injury

**Figure 1:**
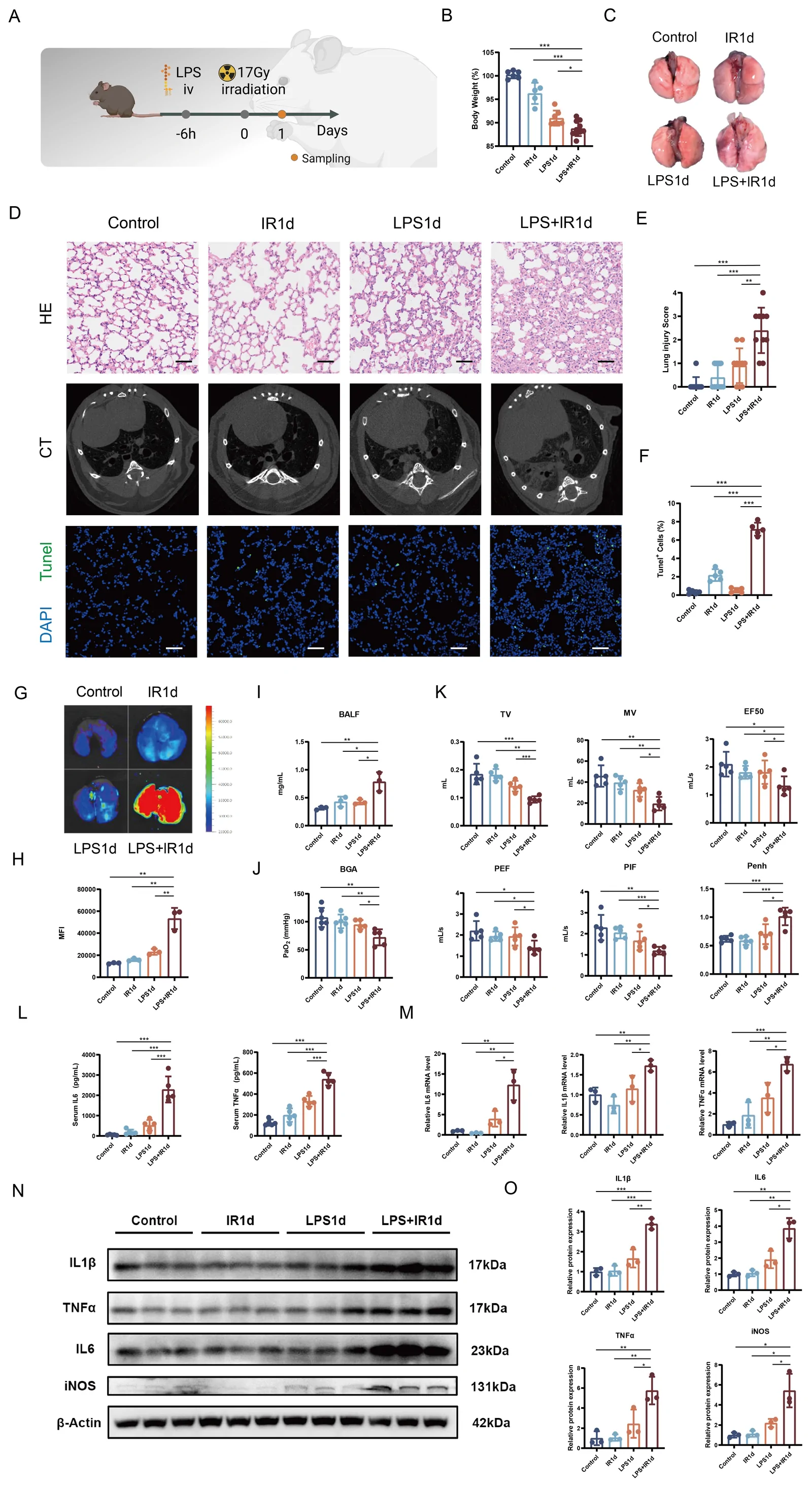
** Combination of LPS and thoracic irradiation exacerbates acute lung injury. (A)** Schematic of the combined lung injury model. **(B-O)** Comparative assessment of acute lung injury at day 1 post-injury among the Control, IR, LPS, and LPS+IR groups.** (B)** Body weight changes (%). **(C)** Representative gross images of lungs.** (D)** Representative images of H&E staining, Micro-CT scans, and TUNEL staining of lung tissues (H&E and TUNEL scale bars, 50 μm).** (E)** Quantification of lung injury scores from H&E staining. **(F)** Quantification of TUNEL-positive cells. **(G)** Assessment of lung vascular permeability via FITC-dextran leakage assay and **(H)** its quantification.** (I)** Quantification of total protein concentration in BALF.** (J)** Quantification of arterial blood gases.** (K)** Lung function parameters at day 1, including MV, TV, PIF, PEF, EF50, and Penh.** (L)** Serum levels of IL-6 and TNF-α measured by ELISA.** (M)** Relative mRNA expression of *Il6*, *Il1β*, and *Tnfα* in lung tissues. **(N-O)** Representative Western blots and corresponding quantification of pro-inflammatory proteins (IL-6, IL-1β, TNF-α, iNOS) in lung tissues. Data are presented as mean ± SD. Statistical significance is indicated as ns, not significant; *P < 0.05, **P < 0.01, ***P < 0.001.

To investigate the synergistic effect of LPS and IR on lung injury, we established a murine model of combined lung injury. Mice were administered graded doses of LPS (2, 5 or 10 mg/kg) via tail vein injection to induce varying degrees of inflammatory stress, followed by 17 Gy whole-thorax X-ray irradiation 6 h later (TNF-α ELISA kit, Figure 1A). Histopathological assessment at day 1 showed that all combined lung injury groups (2 mg/kg LPS+IR, 5 mg/kg LPS+IR, 10 mg/kg LPS+IR) exhibited significantly more severe acute pathology compared to their respective LPS-alone groups (2 mg/kg LPS, 5 mg/kg LPS, 10 mg/kg LPS), the IR-alone group, and the Control group. This synergistic pathology was characterized by severe disruption of lung tissue architecture and prominent inflammatory cell infiltration, accompanied by significantly elevated lung injury scores (Supplemental Figure 1, A and B). Lung function tests further confirmed marked respiratory impairment in the combined injury groups (Supplemental Figure 1C). Notably, the 2 mg/kg LPS combined with irradiation demonstrated the most pronounced synergistic amplification of lung injury relative to the corresponding single-injury groups (2 mg/kg LPS alone and IR alone). Since this dosage induced robust synergistic damage while maintaining high model stability and survival rates, it was selected for subsequent studies.

Using this murine combined lung injury model, we conducted a comprehensive characterization of the acute injury profile at day 1 by comparing the LPS+IR group with the LPS, IR, and Control groups. While the IR and LPS groups showed physiological changes of varying degrees compared with the Control group, the LPS+IR group showed markedly greater systemic and local pathophysiological alterations than either single-injury group. Systemically, the LPS+IR group showed significant weight loss (Figure 1B), accompanied by erythropenia and leukocytosis (Supplemental Figure 2A). Macroscopically, lungs from the LPS+IR group showed extensive congestion and patchy hemorrhage (Figure 1C). Histopathological analysis confirmed that the LPS+IR group exhibited extensive alveolar structural disruption, profuse inflammatory cell infiltration, interstitial edema, and hyaline membrane formation, resulting in lung injury scores significantly higher than those in the Control and single-injury groups (Figure 1, D and E). Micro-computed tomography (Micro-CT) imaging corroborated these findings by revealing diffuse ground-glass opacities, which represent radiological hallmarks of interstitial edema and infiltration that were absent or minimal in the single-injury groups (Figure 1D). Consistent with this severe tissue damage, TUNEL staining indicated a marked increase in cell death in the LPS+IR group (Figure 1, D and F), while immunofluorescence for 8-hydroxy-2'-deoxyguanosine (8-OHdG) and γ-H2AX suggested significantly elevated oxidative stress (Supplemental Figure 3, A-D).

Functionally, these structural deficits resulted in profound respiratory impairment. The LPS+IR group exhibited significant vascular hyperpermeability and protein leakage, as determined by lung permeability assays and BALF protein quantification (Figure 1, G-I). Blood gas analysis showed a significant reduction in arterial oxygen partial pressure (PaO2) in the LPS+IR group (Figure 1J). Moreover, lung function testing revealed a severe restrictive ventilation pattern and elevated airway resistance, evidenced by significant decreases in Minute Ventilation (MV), Tidal Volume (TV), Peak Expiratory Flow (PEF), Peak Inspiratory Flow (PIF), and EF50, with a concomitant increase in Penh (Figure 1K). At the molecular level, the combined injury triggered a dysregulated systemic and local pro-inflammatory response that was heightened relative to the single-injury groups. Systemically, serum enzyme-linked immunosorbent assay (ELISA) results showed significantly elevated circulating levels of IL-6 and TNF-α in the LPS+IR group (Figure 1L). Locally, the mRNA expression of pro-inflammatory cytokines* IL-6*, *IL-1β*, and *TNF-α* was significantly upregulated in the lung tissue of the LPS+IR group (Figure 1M). This trend was validated at the protein level, with Western blot analysis showing markedly increased expression of IL-6, IL-1β, TNF-α, and iNOS (Figure 1, N and O).

### Combination of LPS and Thoracic Irradiation Accelerates Fibrotic Progression

**Figure 2:**
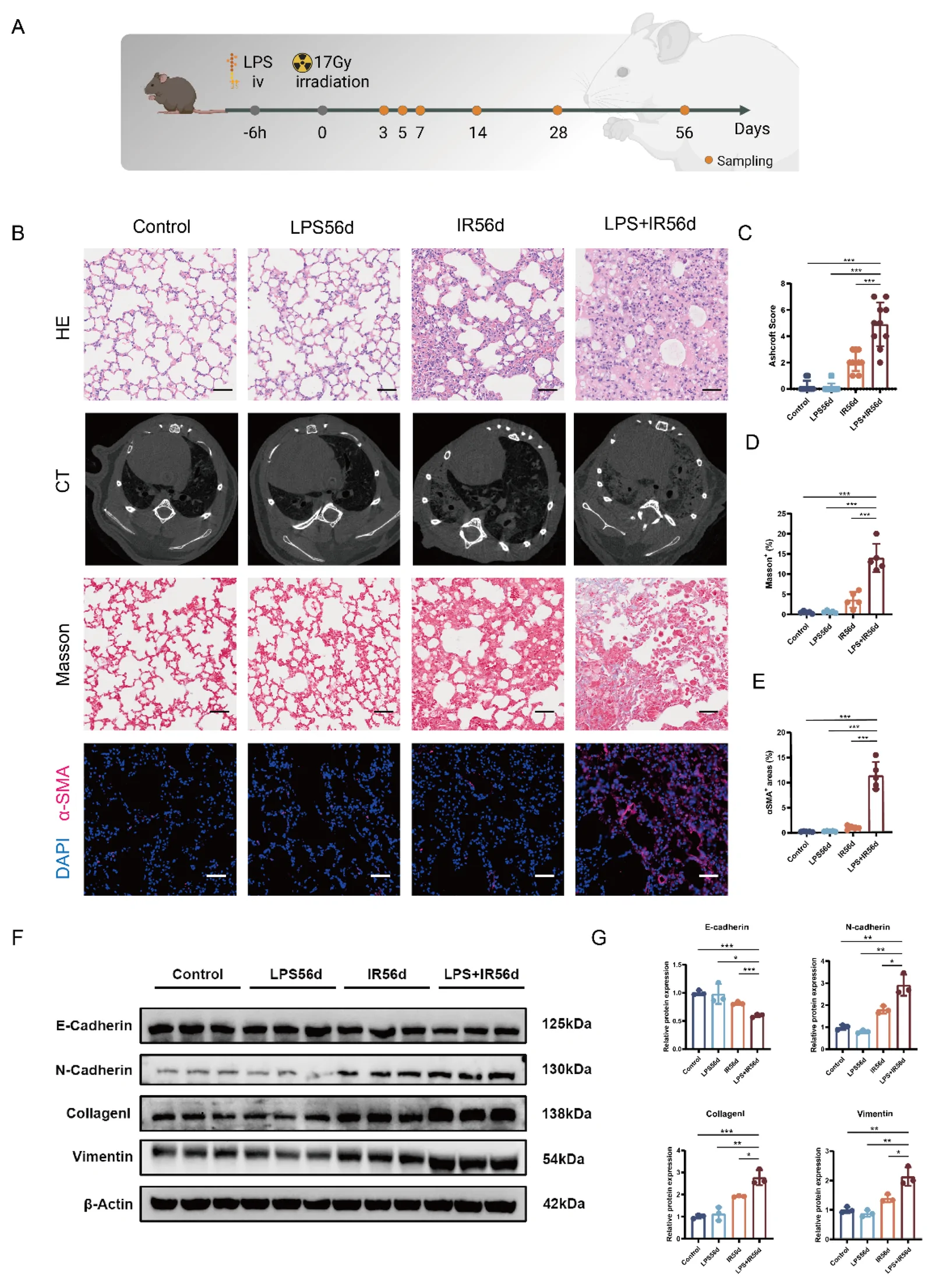
** Combination of LPS and thoracic irradiation accelerates fibrotic progression. (A)** Schematic of the combined lung injury model. **(B-G)** Comparative assessment of late-stage pulmonary fibrosis at day 56 post-injury among the Control, LPS, IR, and LPS+IR groups. **(B)** Representative images of H&E staining, Micro-CT scans, Masson's trichrome staining, and α-SMA immunofluorescence staining of lung tissues (H&E, Masson's trichrome, and α-SMA scale bars, 50 μm). **(C)** Quantification of lung fibrosis using the Ashcroft scoring system.** (D)** Quantification of Masson-positive area. **(E)** Quantification of α-SMA-positive area. **(F-G)** Representative Western blots and corresponding quantification of EMT-related (E-cadherin, N-cadherin) and fibrosis-related (Vimentin, Collagen I) proteins. Data are presented as mean ± SD. Statistical significance is indicated as ns, not significant; *P < 0.05, **P < 0.01, ***P < 0.001.

To delineate the pathological progression, we performed a longitudinal analysis of lung tissues at days 3, 5, 7, 14, 28, and 56 post-modeling (Figure 2A). Histopathological results showed that the IR group developed initial inflammation at day 7, which progressively worsened and led to incipient fibrosis by day 56 (Supplemental Figure 4A). In contrast, acute injury in the LPS group was transient and resolved spontaneously (Supplemental Figure 4C). Both of these findings were consistent with previous reports 19,20. However, the LPS+IR group exhibited the most severe injury trajectory at all time points, transitioning from an early phase (days 3-7) of acute exudative inflammation to a later phase (days 14-56) of accelerated aberrant tissue remodeling and fibrosis. This dynamic progression was also corroborated by serial CT imaging (Supplemental Figure 4, A-G). Compared with the classical murine thoracic irradiation model, in which definitive fibrosis usually develops after 16 weeks or longer 19, the LPS+IR group had already developed a typical fibrotic phenotype by day 56. Histopathological analysis revealed severe alveolar architectural distortion, fibroblastic foci, and extensive collagen deposition on H&E staining, with Ashcroft scores significantly higher than those in the other groups (Figure 2, B and C). Micro-CT imaging displayed classic fibrotic signs, including reticular patterns, linear opacities, and honeycombing (Figure 2B). Consistent with these findings, Masson's trichrome staining showed extensive collagen deposition (Figure 2, B and D). Immunofluorescence staining for α-SMA showed a marked increase in positive areas, indicating myofibroblast activation and accumulation (Figure 2, B and E).

To further validate differences in the severity of pulmonary fibrosis among groups in addition to the histopathological evidence, we employed a molecular tracing approach using IR-780, a fibrosis-targeting tracer previously developed and validated by our group 21,22. IR-780 specifically accumulates in fibrotic lung tissue, and its fluorescence intensity correlates well with the extent of fibrosis. *Ex vivo* imaging at day 56 showed markedly increased IR-780 fluorescence in lungs from the LPS+IR group, with significantly higher mean fluorescence intensity (Supplemental Figure 5, A-B). Consistently, cryosection analysis revealed a broader distribution and a significantly increased positive area of IR-780 signals in the lungs of the LPS+IR group (Supplemental Figure 5, C-D). These findings further support, at the molecular imaging level, a more pronounced late-stage pulmonary fibrotic phenotype in the LPS+IR group.

Given that EMT is a key pathological process involved in aberrant repair and tissue remodeling during the development of pulmonary fibrosis 23, we further evaluated the expression of EMT- and fibrosis-related proteins in lung tissues from each group at the molecular level. Western blot analysis revealed abnormal expression of EMT- and fibrosis-related proteins in the LPS+IR group compared with the other groups, characterized by downregulation of E-cadherin and upregulation of N-cadherin, Collagen I, and Vimentin (Figure 2, F and G).

### Pro-inflammatory Macrophage Infiltration Characterizes the Pathological Process of Combined Lung Injury

**Figure 3:**
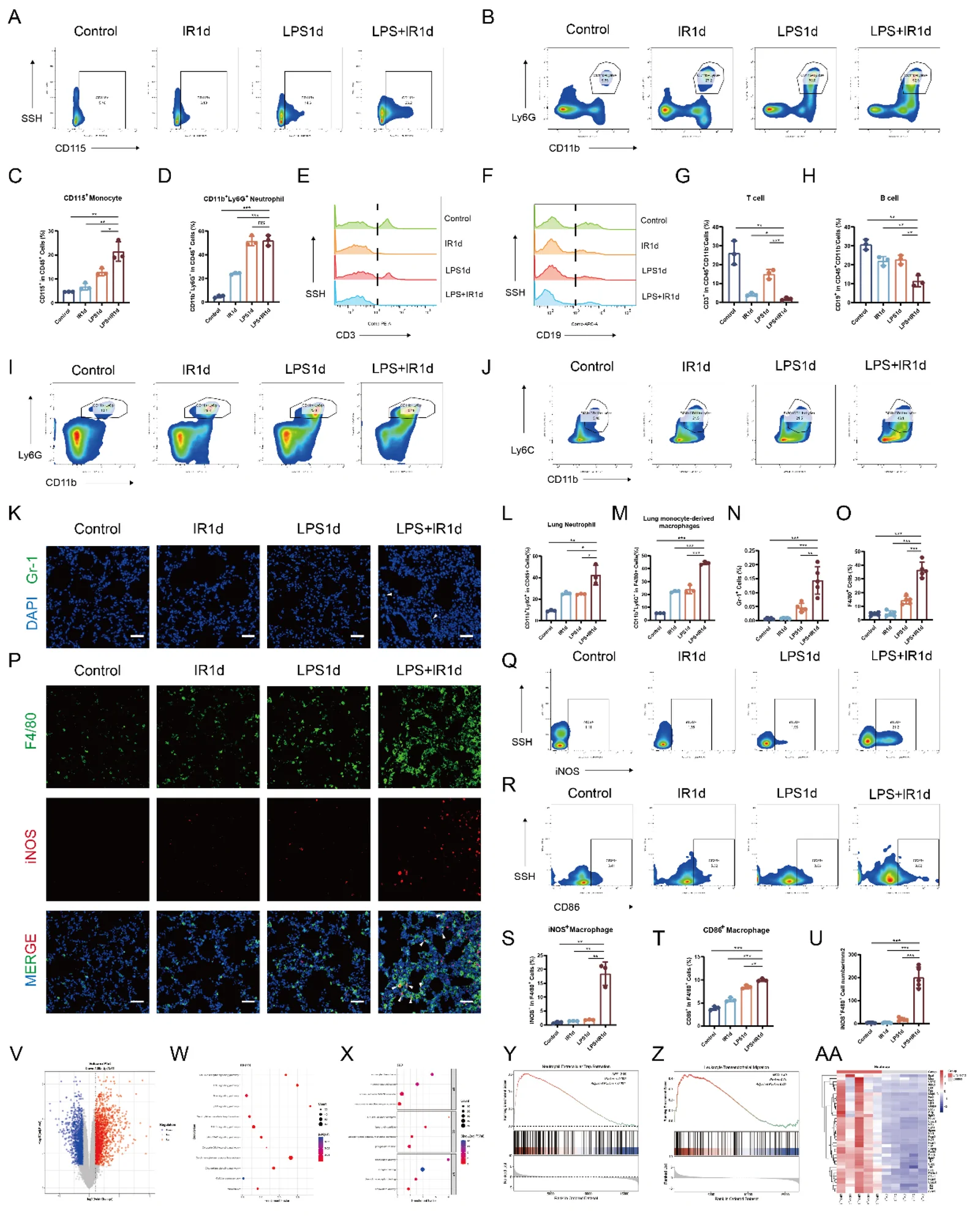
** Assessment of immune cell infiltration and transcriptomic changes at day 1 post-injury. (A-D)** Analysis of circulating immune cells across the four experimental groups (Control, IR, LPS, and LPS+IR) at day 1. **(A)** Representative flow cytometry plots of circulating monocytes (CD45⁺CD11b⁺CD115⁺) and** (B)** neutrophils (CD45⁺CD11b⁺Ly6G⁺). **(C-D)** Quantification of circulating monocyte and neutrophil proportions. **(E-U)** Analysis of lung-infiltrating immune cells and macrophage polarization across the four experimental groups at day 1. **(E)** Representative flow cytometry plots of lung T cells (CD45⁺CD11b⁻CD3⁺) and **(F)** B cells (CD45⁺CD11b⁻CD19⁺). **(G-H)** Quantification of T cell and B cell proportions in the lung. **(I)** Representative flow cytometry plots of lung-infiltrating neutrophils (CD45⁺CD11b⁺Ly6G⁺) and** (J)** monocyte-derived macrophages (CD45⁺F4/80⁺CD11b⁺Ly6C⁺). **(K)** Representative immunofluorescence images of Gr-1⁺ neutrophils in lung tissue (scale bars, 50 μm). **(L-M)** Quantification of neutrophil and monocyte-derived macrophage proportions in lung tissue. **(N-O)** Quantification of Gr-1⁺ and F4/80⁺ cells from immunofluorescence images. **(P)** Representative immunofluorescence co-staining of F4/80 and iNOS (scale bars, 50 μm). **(Q)** Representative flow cytometry plots of M1-polarized macrophages (CD11b⁺F4/80⁺iNOS⁺) and **(R)** (CD11b⁺F4/80⁺CD86⁺). **(S-T)** Quantification of iNOS⁺ and CD86⁺ M1 macrophage proportions. **(U)** Quantification of F4/80⁺iNOS⁺ double-positive cells. **(V-AA)** Transcriptomic analysis of lung tissue from LPS+IR and Control groups at day 1. **(V)** Volcano plot of differentially expressed genes (DEGs). **(W)** KEGG pathway and **(X)** GO functional enrichment analyses of DEGs. **(Y)** GSEA plot showing enrichment of the "Neutrophil extracellular trap formation" pathway. **(Z)** GSEA plot showing enrichment of the "Leukocyte transendothelial migration" pathway. **(AA)** Heatmap of ETosis-related genes. Data are presented as mean ± SD. Statistical significance is indicated as ns, not significant; *P < 0.05, **P < 0.01, ***P < 0.001.

To elucidate the severe pathological features of the combined injury, we performed a comprehensive analysis of the immune landscape by flow cytometry at day 1 after modeling. The results demonstrated that the combined injury triggered remodeling of the immune microenvironment in both peripheral blood and lung tissues. In the periphery, we observed a marked expansion of the myeloid lineage, evidenced by significantly elevated counts of monocytes (CD45⁺CD11b⁺CD115⁺ cells) and neutrophils (CD45⁺CD11b⁺Ly6G⁺ cells) compared with the single-injury and control groups (Figure 3, A-D).

Accompanying the systemic response, analysis of the pulmonary infiltrates revealed a marked reduction in adaptive immune cells. Both T cells (CD45⁺CD11b⁻CD3⁺ cells) and B cells (CD45⁺CD11b⁻CD19⁺ cells) were significantly reduced in the LPS+IR group (Figure 3, E-H). In contrast, myeloid cells, particularly neutrophils (CD45⁺CD11b⁺Ly6G⁺ cells) and monocyte-derived macrophages (F4/80⁺CD11b⁺Ly6C⁺ cells), were significantly enriched in lung tissue (Figure 3, I-J and L-M). Spatially, immunofluorescence staining refined these findings. While flow cytometry confirmed that neutrophil recruitment was significantly elevated in the LPS+IR group, tissue imaging revealed that their absolute abundance within the lung interstitium remained sparse (Figure 3, K and N). In sharp contrast, F4/80⁺ macrophages exhibited dense and diffuse accumulation, characterizing them as the predominant infiltrating population (Figure 3, O and P). These findings identify the massive infiltration of peripheral monocytes that differentiate into macrophages as an important pathological feature of the combined injury.

To define the functional phenotype of these infiltrating macrophages, we assessed their polarization status. Flow cytometry analysis revealed a significant shift toward a pro-inflammatory M1 phenotype in the LPS+IR group, with the expression of M1 markers (CD86, iNOS, CD80) significantly upregulated compared with the single-injury and control groups (Figure 3, Q-T; Supplemental Figure 6, A and B). In contrast, the M2 marker CD301 exhibited only a non-significant upward trend (Supplemental Figure 6, C and D). This pro-inflammatory polarization was further corroborated by immunofluorescence, which showed a profuse accumulation of iNOS⁺F4/80⁺ M1 macrophages in the LPS+IR group (Figure 3, P and U).

To unravel the underlying molecular mechanisms driving this synergistic pathology, we performed RNA-seq analysis on lung tissues at day 1 after modeling. Compared with the Control group, a total of 2936 differentially expressed genes were identified in the combined injury group, of which 1541 were upregulated and 1395 were downregulated (Figure 3V). KEGG and GO pathway enrichment analyses provided a molecular explanation for the immune landscape while uncovering novel pathogenic mechanisms. Consistent with the pro-inflammatory macrophage infiltration observed *in vivo*, pathways such as "Monocyte chemotaxis" and "Macrophage activation" were significantly enriched. Additionally, pathways mediating the crosstalk between immune and endothelial cells, such as "Endothelial cell chemotaxis" and "Leukocyte transendothelial migration", were also prominently identified (Figure 3, W and X). GSEA confirmed that the "Leukocyte transendothelial migration" pathway was significantly activated in the combined injury group (NES = 1.49, adj.P < 0.05) (Figure 3, Z). These transcriptomic signatures suggested that aberrant endothelial activation may serve as a potential upstream event facilitating the massive immune infiltration. Furthermore, the analysis highlighted potential upstream pathways, including "Neutrophil extracellular trap formation", "MAPK signaling pathway", "p53 signaling pathway" and "Cellular senescence".

Finally, to dissect the molecular basis of the synergy, we compared the combined injury group with each single-injury group. Compared to the LPS group, a total of 1257 differentially expressed genes were identified in the combined injury group, of which 699 were upregulated and 558 were downregulated (Supplemental Figure 7A). These DEGs were significantly enriched in pathways such as "NF-kappa B signaling pathway", "p53 signaling pathway", and "Reactive oxygen species metabolic process" (Supplemental Figure 7, B and C). Compared to the IR group, a total of 2872 differentially expressed genes were identified in the combined injury group, of which 1656 were upregulated and 1216 were downregulated (Supplemental Figure 7D). These DEGs were significantly enriched in pathways such as "Toll-like receptor signaling pathway" and "Monocyte chemotaxis" (Supplemental Figure 7, E and F).

In summary, relative to irradiation alone, LPS pretreatment primed and enhanced the innate immune response to radiation injury. Relative to LPS alone, irradiation amplified LPS-induced inflammatory and cellular stress responses, as well as cellular senescence. Together, these findings further support the proposed two-hit synergy model.

### Macrophage Extracellular Traps Formation Serves as an Important Pathogenic Process in Combined Lung Injury

Given that excessive extracellular trap formation (ETosis) is a key driver of acute lung injury 24 and our transcriptomic analysis revealed a significant enrichment of the "Neutrophil extracellular trap formation" pathway, we investigated the role of ETosis in our model. Since current pathway databases lack a general "ETosis" or a specific "METosis" entry, our analysis utilized the "Neutrophil extracellular trap formation" pathway as a functional surrogate. GSEA confirmed that this pathway was significantly activated in the combined injury group (NES = 2.09, adj.P < 0.001) (Figure 3, Y), with the corresponding gene expression pattern shown in the heatmap (Figure 3, AA).

**Figure 4:**
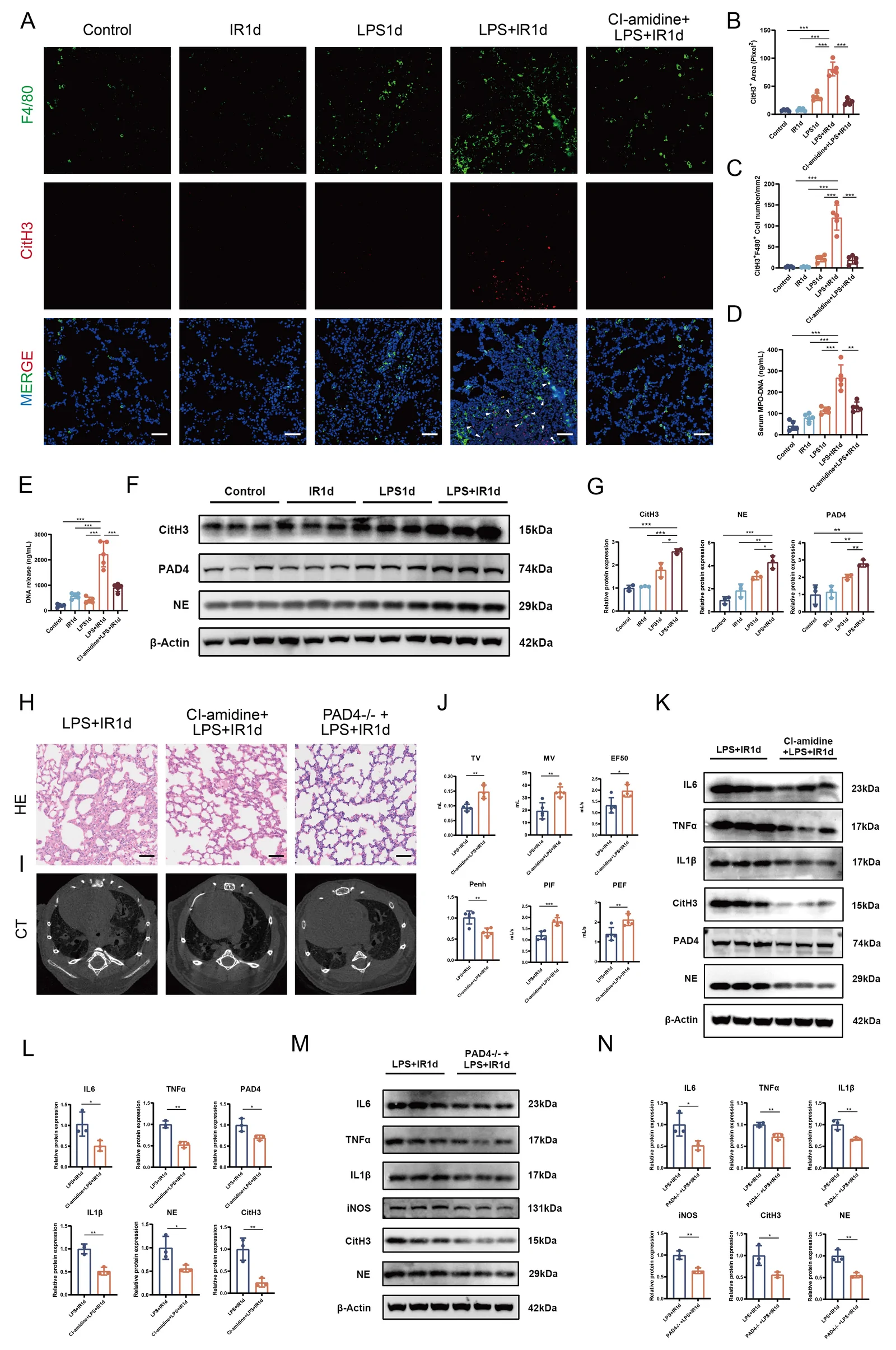
** Macrophage extracellular traps are a key pathogenic mechanism in combined lung injury. (A)** Representative immunofluorescence co-staining of F4/80 and CitH3 in lung tissue at day 1 from the Control, IR, LPS, LPS+IR, and Cl-amidine + LPS+IR groups (scale bars, 50 μm). **(B-C)** Quantification of CitH3 fluorescence intensity and F4/80⁺CitH3⁺ double-positive cells. **(D)** Serum levels of MPO-DNA complexes measured by ELISA at day 1 and **(E)** serum levels of DNA measured by PicoGreen assay at day 1.** (F-G)** Representative Western blots and corresponding quantification of ETosis-related proteins (CitH3, PAD4, NE) in lung tissue at day 1.** (H-N)** Pharmacological and genetic inhibition of PAD4-dependent extracellular trap formation alleviates combined lung injury at day 1. **(H-I)** Representative **(H)** H&E staining and **(I)** Micro-CT images of lungs at day 1 from mice treated with Cl-amidine or from PAD4⁻/⁻ mice following combined injury (H&E scale bars, 50 μm). **(J)** Lung function assessment after Cl-amidine treatment at day 1, including MV, TV, PIF, PEF, EF50, and Penh. **(K-L)** Representative Western blots and corresponding quantification of METosis-related (CitH3, PAD4, NE) and pro-inflammatory proteins (IL-6, IL-1β, TNF-α) in the lungs of Cl-amidine-treated mice following combined injury at day 1. **(M-N)** Representative Western blots and corresponding quantification of METosis-related (CitH3, NE) and pro-inflammatory proteins (IL-6, IL-1β, TNF-α, iNOS) in the lungs of PAD4⁻/⁻ mice following combined injury at day 1. Data are presented as mean ± SD. Statistical significance is indicated as ns, not significant; *P < 0.05, **P < 0.01, ***P < 0.001.

To experimentally validate the activation of ETosis, we first assessed the general extent of ETosis by staining for citrullinated histone H3 (CitH3), a hallmark of chromatin decondensation 25. The results showed a significant increase in CitH3-positive areas in the LPS+IR group, indicating enhanced release of extracellular chromatin (Figure 4, A and B). To distinguish the specific cellular origin of these extracellular traps (ETs), we subsequently performed co-localization studies using cell-specific markers. Notably, we identified macrophages as the predominant source, evidenced by a significant accumulation of CitH3⁺F4/80⁺ double-positive cells in the LPS+IR group (Figure 4, A and C). To mechanistically corroborate the occurrence of METosis, we examined peptidyl arginine deiminase 4 (PAD4), a core downstream effector involved in ETosis. Immunofluorescence analysis revealed that the number of PAD4⁺F4/80⁺ double-positive cells was significantly increased in the LPS+IR group (Supplemental Figure 8, A and C). Conversely, despite the classical association of ETosis with neutrophils, the frequency of CitH3⁺Ly6G⁺ double-positive cells were negligible and showed no significant intergroup differences (Supplemental Figure 9, A and C). These findings collectively suggest that METosis is the dominant form of ETosis in the pathology of combined injury.

To further validate this phenomenon, we measured related systemic and molecular markers of ETosis. Systemic levels of cell-free DNA and myeloperoxidase (MPO)-DNA complexes in the peripheral blood were significantly elevated in the LPS+IR group, supporting the occurrence of ETosis at the systemic level (Figure 4, D and E). Consistently, Western blot analysis showed that the expression of METosis-related proteins CitH3, PAD4, and neutrophil elastase (NE) was significantly upregulated in lung tissue from the LPS+IR group (Figure 4, F and G).

Since PAD4-mediated histone citrullination is considered a critical step in driving chromatin decondensation and extracellular trap formation, targeting PAD4 represents an important strategy for inhibiting METosis 26. To establish the pathogenic role of METosis, we employed both pharmacological inhibition and genetic knockout strategies. First, treatment with the PAD4 inhibitor Cl-amidine significantly attenuated combined injury-induced histopathological damage and radiological abnormalities (Figure 4, H and I), while also improving lung function by alleviating restrictive ventilation dysfunction and reducing airway resistance (Figure 4J). Mechanistically, Cl-amidine treatment effectively reduced the number of pulmonary CitH3⁺F4/80⁺ cells (Figure 4, A and C) and significantly downregulated the expression of both pro-inflammatory proteins (IL-6, TNF-α, IL-1β) and METosis-related proteins (CitH3, PAD4, NE) (Figure 4, K and L). Second, we replicated these findings in PAD4 knockout mice, where the genetic absence of PAD4 conferred similar protection against acute combined lung injury (Figure 4, H and I) and significantly suppressed the expression of inflammatory (IL-6, TNF-α, IL-1β, iNOS) and METosis-related proteins (CitH3, NE) (Figure 4, M and N). Taken together, this evidence supports METosis as an important pathogenic mechanism contributing to the synergistic lung injury caused by LPS and irradiation.

### Stress-Induced Endothelial Senescence Emerges as an Early Feature of Combined Lung Injury

**Figure 5:**
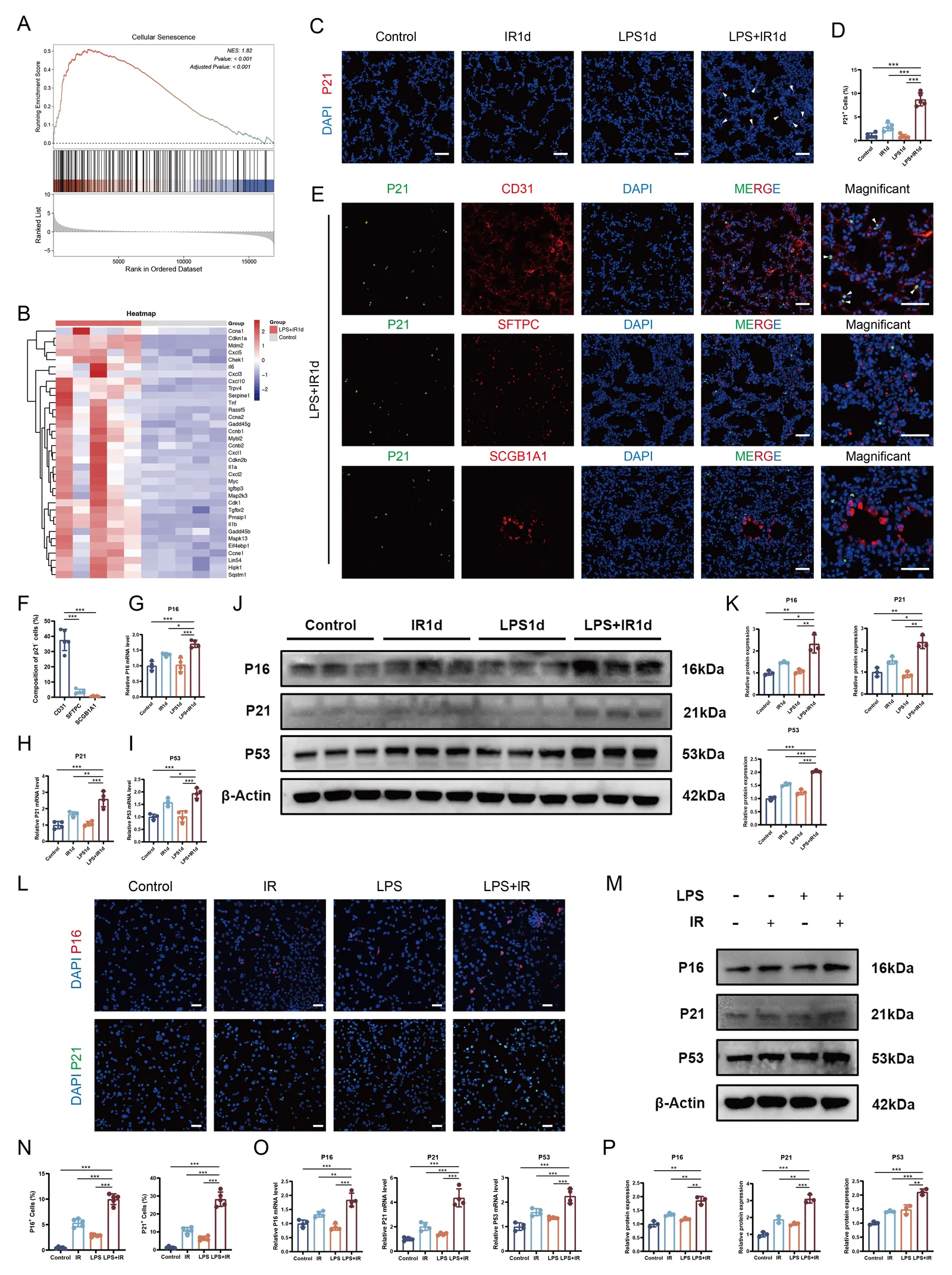
** The combination of LPS and irradiation induces an early endothelial senescence-associated phenotype *in vivo* and *in vitro*. (A)** GSEA plot showing the significant enrichment of the "Cellular Senescence" pathway in the LPS+IR group. **(B)** Heatmap of representative senescence-associated genes across the Control, IR, LPS, and LPS+IR groups at day 1.** (C)** Representative immunofluorescence images of the senescence-associated marker P21 in lung tissues at day 1 (scale bars, 50 μm). **(D)** Quantification of P21-positive cells in the lung. **(E)** Representative immunofluorescence co-staining images of P21 with CD31, SFTPC, or SCGB1A1 in lung from the LPS+IR group at day 1 (scale bars, 50 μm). **(F)** Quantification of the proportion of P21-positive cells that co-localize with CD31, SFTPC, or SCGB1A1 from (E).** (G-I)** Relative mRNA expression of *p16*, *p21*, and *p53* in lung tissues at day 1.** (J)** Representative Western blots and **(K)** quantitative analysis of senescence-related proteins (P16, P21, P53) in lung tissues at day 1.** (L)** Representative immunofluorescence images of senescence-related markers P16 and P21 in cultured endothelial cells (scale bars, 100 μm).** (M)** Representative Western blots of cellular senescence-related proteins (P16, P21, P53) in endothelial cells.** (N)** Quantification of P16- and P21-positive cells from (L).** (O)** Relative mRNA expression of *p16*, *p21*, and *p53* in endothelial cells.** (P)** Quantification of senescence-related proteins (P16, P21, P53) from (M). Data are presented as mean ± SD. Statistical significance is indicated as ns, not significant; *P < 0.05, **P < 0.01, ***P < 0.001.

Having established METosis as an important pathogenic process in combined lung injury, we next sought to identify the upstream cellular state that initiates or amplifies this response. Transcriptomic profiling revealed a prominent senescence-associated signature in the LPS+IR group, as evidenced by GSEA enrichment of the cellular senescence pathway and coordinated upregulation of senescence-associated genes in the heatmap (Figure 5, A and B). Building on this evidence, and based on the report by Binet *et al*. showing that a senescence-associated secretome can promote NETosis 27, we hypothesized that the combination of LPS and irradiation induces an early senescence-associated program in the lung, potentially establishing a pro-inflammatory secretory milieu that could shape macrophage responses and facilitate METosis. To test this hypothesis *in vivo*, we first validated these transcriptomic findings at the tissue level and confirmed the emergence of an early senescence-associated phenotype in the lung.

Immunofluorescence staining showed increased nuclear p21 signals in the LPS+IR group at day 1 compared with the Control, LPS, and IR groups (Figure 5, C and D). Consistently, immunofluorescence staining also showed increased nuclear signals of p16 and p53 in the LPS+IR group (Supplemental Figure 10, A and B). To pinpoint the cellular origin of this early senescence signal, we performed multiplex immunofluorescence and observed that p21-positive cells predominantly colocalized with the endothelial marker CD31, with substantially less overlap with epithelial markers SFTPC and SCGB1A1, supporting endothelial cells as the principal cellular source of the early senescence-associated changes after combined injury (Figure 5, E and F). Consistent with the staining results, qPCR and Western blot analyses of lung tissues showed significant upregulation of p16, p21, and p53 in the LPS+IR group (Figure 5, G-K).

To further substantiate our *in vivo* observations, we exposed cultured endothelial cells to combined LPS and irradiation stimulation and observed a concordant senescence-associated phenotype. Immunofluorescence staining demonstrated a marked nuclear accumulation of p16 and p21 in LPS+IR-treated endothelial cells (Figure 5, L and N). In parallel, qPCR and Western blot analyses confirmed significant upregulation of p16, p21, and p53 at both the mRNA and protein levels in LPS+IR-treated endothelial cells (Figure 5, M and O-P). Consistently, SA-β-gal staining showed that the proportion of SA-β-gal-positive endothelial cells was already increased in the LPS+IR group at 24 h compared with the Control, LPS, and IR groups, and this increase became more pronounced at 48 h (Supplemental Figure 11, A-D). Collectively, these data suggest that the synergistic stress of LPS and irradiation induces early senescence-associated alterations in endothelial cells within 24 h, providing a potential cellular basis for subsequent paracrine signaling that acts on macrophages.

### Endothelial Senescence-Associated Secretory Profile Promotes Macrophage Extracellular Traps Formation

**Figure 6:**
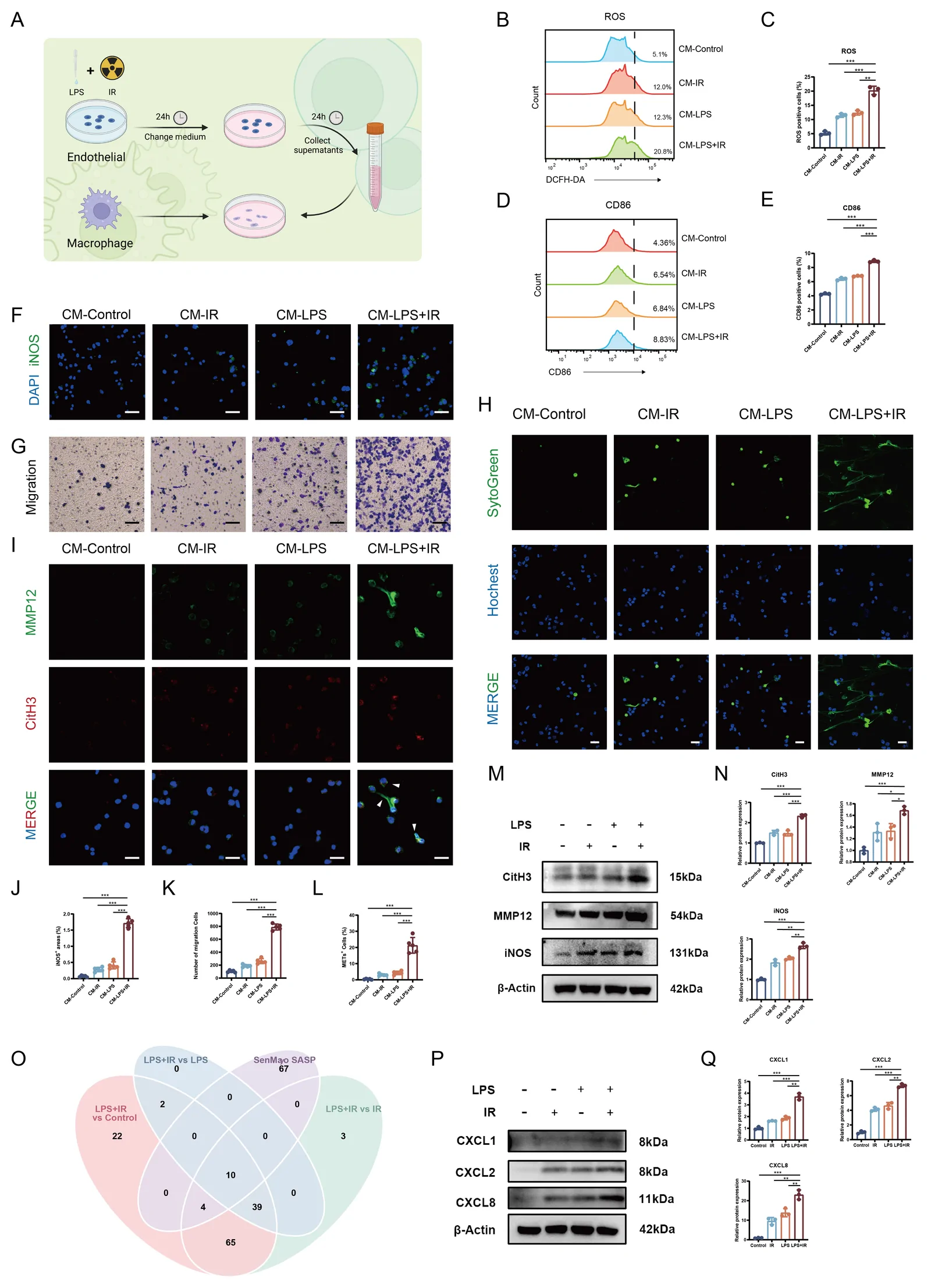
** Endothelial senescence-associated secretory profile promotes macrophage extracellular traps formation. (A)** Schematic of the endothelial-macrophage co-culture system. **(B-N)** Macrophages were treated with conditioned medium (CM) from four groups of endothelial cells: untreated (Control), irradiated (IR), LPS-treated (LPS), or co-stimulated with LPS and IR (LPS+IR). **(B)** Representative flow cytometry plots of macrophage ROS production in each group. **(C)** Quantification of ROS-positive cells from (B). **(D)** Representative flow cytometry plots of macrophage M1 polarization (CD86) in each group. **(E)** Quantification of CD86-positive cells from (D). **(F)** Representative immunofluorescence images of the M1 marker iNOS (scale bars, 200 μm).** (G)** Representative images of macrophage migration assays (scale bars, 200 μm). **(H)** Representative immunofluorescence images of METs (SytoGreen/Hoechst staining) (scale bars, 100 μm). **(I)** Representative immunofluorescence co-staining of CitH3 and MMP12 (scale bars, 200 μm). **(J)** Quantification of iNOS-positive area.** (K)** Quantification of migrated macrophages. **(L)** Quantification of METs-positive cells. **(M-N)** Representative Western blots and corresponding quantification of METosis-related proteins (CitH3, MMP12) and pro-inflammatory proteins (iNOS) in macrophages.** (O)** Venn diagram showing the overlap of commonly upregulated genes in the LPS+IR group compared with the Control, LPS, and IR groups with the SenMayo SASP gene set. **(P-Q)** Representative Western blots and corresponding quantification of SASP-related CXC chemokines (CXCL1, CXCL2, CXCL8) in endothelial cells. Data are presented as mean ± SD. Statistical significance is indicated as ns, not significant; *P < 0.05, **P < 0.01, ***P < 0.001.

To further investigate how endothelial cells with senescence-associated alterations regulate macrophage responses, we established an *in vitro* endothelial-macrophage co-culture system. Macrophages were exposed to conditioned medium (CM) collected from endothelial cells previously stimulated with LPS, IR, or LPS+IR (Figure 6A). The results showed that the endothelial secretome derived from the co-stimulated group proved to be a potent driver of macrophage activation, inducing a response significantly stronger than that of the Control or single-stimulus groups. This was demonstrated by flow cytometry analysis showing increased reactive oxygen species (ROS) production and upregulated expression of the M1 marker CD86 (Figure 6, B-E), while immunofluorescence staining confirmed an increased number of iNOS⁺ M1 macrophages (Figure 6, F and J). Additionally, cell migration was significantly enhanced (Figure 6, G and K). Crucially, this inflammatory secretome acted as a robust inducer of METosis. SytoGreen/Hoechst staining showed that macrophages treated with CM-LPS+IR exhibited thin, fibrous, and interwoven web-like structures formed by extracellular DNA release (Figure 6, H and L). Multiplex immunofluorescence further revealed enhanced colocalization of matrix metalloproteinase 12 (MMP12) with CitH3 within these filamentous structures (Figure 6I). Western blot analysis further confirmed that the expression of METosis-related proteins (CitH3, MMP12) and the M1 marker iNOS were all significantly upregulated in these macrophages (Figure 6, M and N). Similar effects were observed in a co-culture system using primary murine cells (Supplemental Figure 12, A and B).

Having observed the functional impact of the endothelial secretome on macrophage activation and METosis, we next sought to identify the candidate effector molecules in the senescence-associated secretory profile that mediate METosis. Accordingly, endothelial cells treated under the Control, IR, LPS, and LPS+IR conditions were collected at 24 h for RNA sequencing. Differentially expressed genes in the LPS+IR group were then compared relative to the Control, LPS, and IR groups, respectively, and the commonly upregulated genes were intersected with the SenMayo SASP gene set. Through this intersection analysis, 10 overlapping genes were identified (Figure 6O), including CXCL1, IL6, PAPPA, CXCL2, CXCL3, CXCL8, CSF2, C3, IL1B, and CCL20.

Among these overlapping genes, CXC chemokines were particularly prominent, notably CXCL1, CXCL2, CXCL3, and CXCL8, suggesting that they may serve as important candidate components of the endothelial secretory profile induced by the combined stimulation. To further validate these transcriptomic findings at the protein level, we performed a cytokine array analysis of endothelial cell-conditioned media from the Control, LPS, IR, and LPS+IR groups. Consistent with the RNA-seq results, CXCL1, CXCL2, CXCL3, and CXCL8 were markedly elevated in the LPS+IR group compared with the Control and single-stimulus groups (Supplemental Figure 13, A and B). Western blot analysis further confirmed that combined LPS and IR stimulation induced significant upregulation of key SASP-related chemokines, including CXCL1, CXCL2, and CXCL8 (Figure 6, P and Q).

As DAMPs released from damaged cells are also known to promote ETosis 28, we further performed an exclusion experiment to assess the potential influence of endothelial cell death. Endothelial cells were treated with the pan-caspase inhibitor Z-VAD-FMK during LPS+IR stimulation, and the conditioned medium was subsequently collected and applied to macrophages. Under this condition, the conditioned medium retained its ability to induce METosis, with no significant difference in METs-positive cells observed after Z-VAD-FMK treatment (Supplemental Figure 14, A and B).

Together, these findings indicate that the endothelial secretome induced by combined LPS and IR stimulation promotes macrophage activation and METosis. This effect is associated with a CXC chemokine-enriched SASP profile rather than being solely attributable to endothelial cell death, supporting a potential endothelial senescence-associated secretory signaling mechanism that promotes macrophage activation and METosis.

### The CXCR2-ROS Axis Mediates the Induction of Macrophage Extracellular Traps

**Figure 7:**
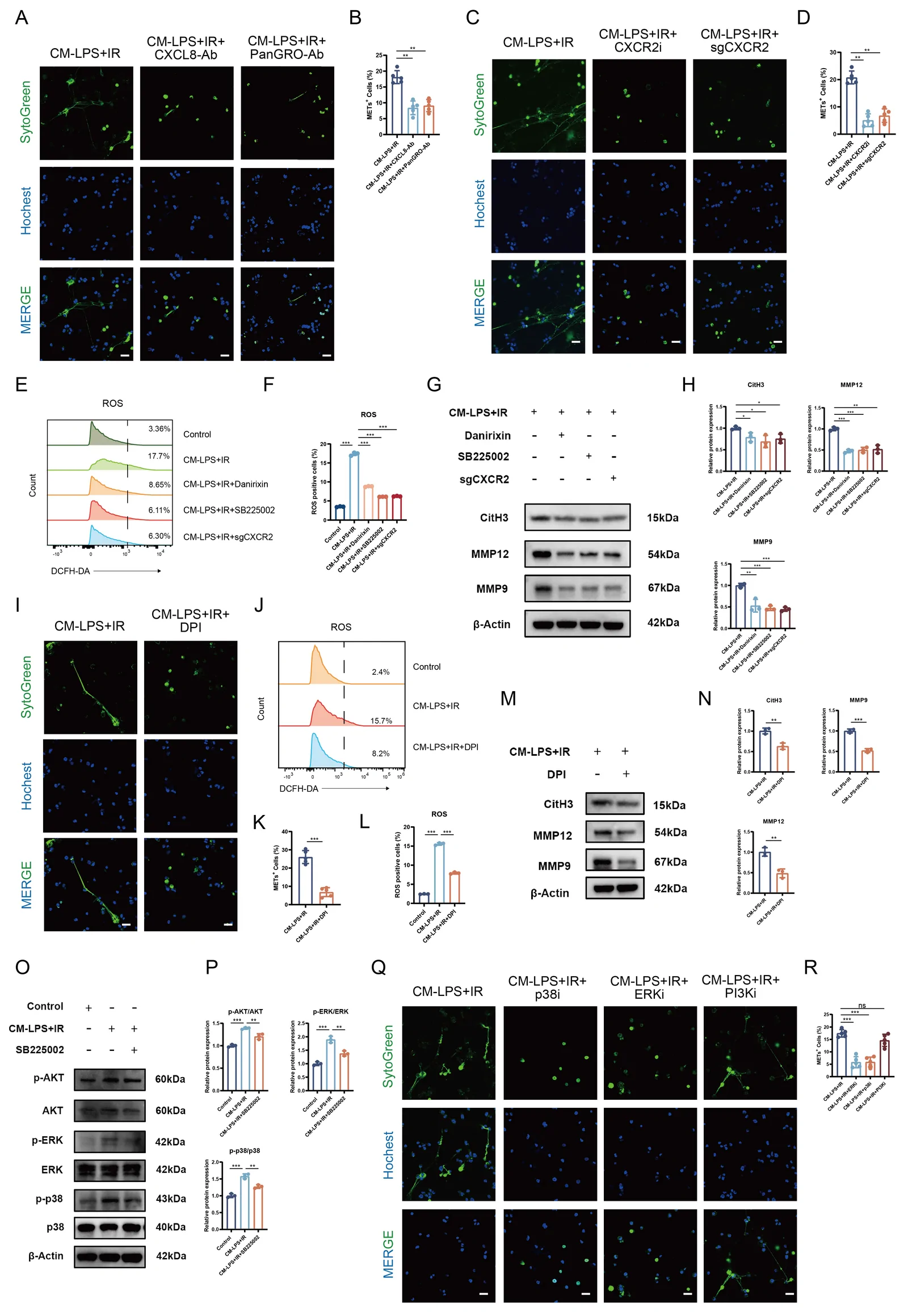
** The CXCR2-ROS axis mediates the induction of macrophage extracellular traps. (A)** Representative immunofluorescence images of METs formation (SytoGreen/Hoechst staining) induced by conditioned medium (CM) from LPS+IR-stimulated endothelial cells, following intervention with anti-CXCL8 or pan-GRO neutralizing antibodies (scale bars, 100 μm). **(B)** Quantification of METs-positive cells from (A). **(C-H)** The CXCR2 axis is involved in METosis. **(C)** Representative immunofluorescence images of METs formation (SytoGreen/Hoechst staining) induced by conditioned medium (CM) from LPS+IR-stimulated endothelial cells, following intervention with CXCR2 inhibitors, or in CXCR2 knockdown cells (scale bars, 100 μm).** (D)** Quantification of METs-positive cells from (C).** (E)** Flow cytometry plots of ROS production in macrophages under the same intervention conditions. **(F)** Quantification of ROS-positive cells from (E). **(G-H)** Representative Western blots and corresponding quantification of METosis-related proteins (CitH3, MMP12, MMP9) under the same intervention conditions. **(I-N)** Role of NADPH oxidase-derived ROS in METosis. **(I-J)** Representative immunofluorescence images of **(I)** METs formation (SytoGreen/Hoechst staining) and **(J)** ROS in macrophages induced by conditioned medium (CM) from LPS+IR-stimulated endothelial cells, following intervention with the NADPH oxidase inhibitor DPI (scale bars, 100 μm). **(K-L)** Quantification of METs-positive cells and ROS-positive cells from (I-J).** (M-N)** Representative Western blots and corresponding quantification of METosis-related proteins (CitH3, MMP12, MMP9) under the same intervention conditions. **(O-R)** Downstream signaling pathways involved in CM-LPS+IR-induced METosis. **(O-P)** Representative Western blots and corresponding quantification of p-AKT/AKT, p-ERK/ERK, and p-p38/p38 in macrophages stimulated with CM from LPS+IR-treated endothelial cells, with or without SB225002 treatment. **(Q-R)** Effects of p38, ERK, and PI3K inhibition on CM-LPS+IR-induced METosis. **(Q)** Representative immunofluorescence images of METs formation (SytoGreen/Hoechst staining) in macrophages under the indicated conditions (scale bars, 100 μm). **(R)** Quantification of METs-positive cells from (Q). Data are presented as mean ± SD. Statistical significance is indicated as ns, not significant; *P < 0.05, **P < 0.01, ***P < 0.001.

To determine whether SASP-related chemokines contribute to METosis, we first performed neutralization experiments using an anti-CXCL8 neutralizing antibody and a pan-GRO neutralizing antibody. Notably, blocking either CXCL8 or CXCL1/2/3 chemokines attenuated METosis (Figure 7, A and B). Since CXCL1, CXCL2, CXCL3, and CXCL8 primarily exert their biological effects through CXCR2, we next investigated the central role of CXCR2 in endothelial senescence-associated secretory signaling-induced METosis. We first generated a CXCR2-knockdown macrophage cell line using CRISPR-Cas9 (Supplemental Figure 15, A-E). In parallel, we utilized CXCR2 inhibitors (SB225002 and Danirixin). Both genetic knockdown and pharmacological inhibition of CXCR2 consistently attenuated CM-LPS+IR-induced METosis (Figure 7, C and D), reduced intracellular ROS levels (Figure 7, E and F), and downregulated the expression of METosis-related proteins (CitH3, MMP12, MMP9) (Figure 7, G and H). Together, these results support CXCR2 as an important signaling node through which endothelial senescence-associated secretory signaling promotes macrophage METosis.

ROS is an important upstream signal involved in extracellular trap formation 29. To determine whether ROS generation contributes to CM-LPS+IR-induced METosis, macrophages were treated with DPI, an NADPH oxidase inhibitor. This intervention attenuated CM-LPS+IR-induced METosis (Figure 7, I and K), reduced intracellular ROS levels (Figure 7, J and L), and downregulated the expression of METosis-related proteins (CitH3, MMP12, MMP9) (Figure 7, M and N). These findings indicate that NADPH oxidase-dependent ROS generation contributes to CM-LPS+IR-induced METosis.

We then investigated the downstream intracellular pathways linking CXCR2 activation to ROS production and METosis. Previous studies have shown that CXCR2 can activate multiple signaling pathways, including p38, ERK, and PI3K/AKT 30. Consistent with this, Western blot analysis showed that CM-LPS+IR stimulation increased the phosphorylation levels of p38, ERK, and AKT in macrophages, whereas these changes were attenuated after CXCR2 inhibition (Figure 7, O and P). To further explore the functionally relevant downstream pathways, we used pharmacological inhibitors targeting p38, ERK, and PI3K. Inhibition of either p38 or ERK attenuated CM-LPS+IR-induced METosis, whereas PI3K inhibition had little effect (Figure 7, Q and R). These results suggest that CXCR2 promotes ROS generation and METosis through the involvement of p38 and ERK signaling pathways.

### Macrophage Extracellular Traps Contribute to Epithelial Barrier Disruption and Epithelial-Mesenchymal Transition

Emerging evidence suggests that ETosis exerts multifaceted pathological effects, functioning as an important mediator of intercellular crosstalk that modulates bystander cells 31,32. To investigate the specific functional impact of METs on pulmonary epithelial cells, we established a macrophage-epithelial cell co-culture model. To generate METs for epithelial cell stimulation, we first screened the candidate SASP-associated chemokines identified in our analysis, including CXCL1, CXCL2, CXCL3, and CXCL8. Among them, CXCL8 induced the most pronounced METosis (Supplemental Figure 16, A and B). We therefore used recombinant CXCL8 in subsequent experiments to induce METosis and generate conditioned medium for epithelial cell treatment.

**Figure 8:**
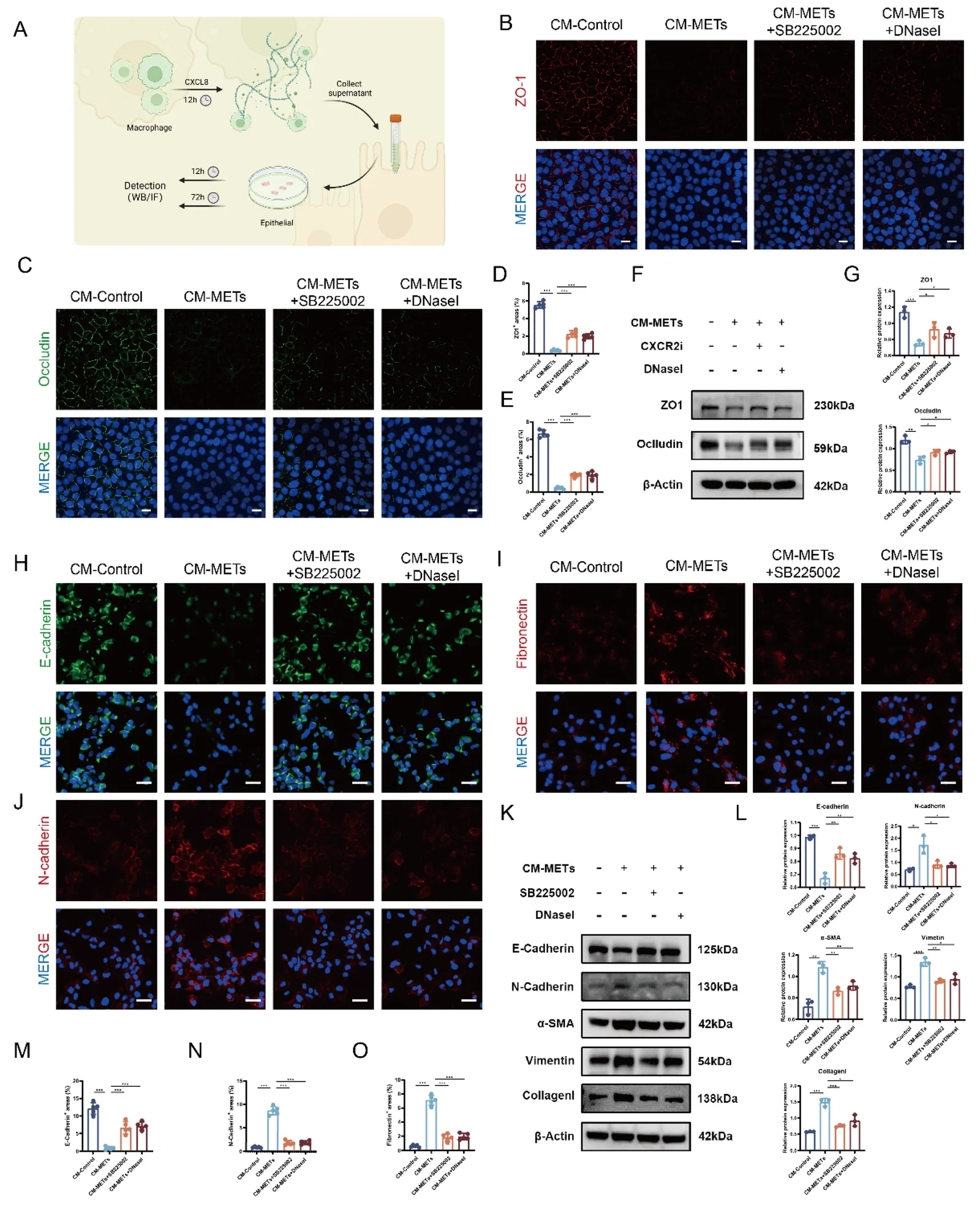
** Macrophage extracellular traps induce early epithelial barrier disruption and late-stage EMT-like changes. (A)** Schematic of the macrophage-epithelial co-culture system. **(B-G)** Early-stage (12 h) epithelial barrier disruption induced by METs and its attenuation by intervention.** (B-C)** Representative immunofluorescence images of tight junction proteins **(B)** ZO-1 and **(C)** Occludin in epithelial cells treated for 12 h with CM-METs with or without interventions. Interventions included a CXCR2 inhibitor to block METs formation or DNase I to degrade METs structures (scale bars, 50 μm).** (D-E)** Quantification of fluorescence intensity for ZO-1 and Occludin. **(F-G)** Representative Western blots and corresponding quantification of ZO-1 and Occludin under the same treatment conditions. **(H-O)** Late-stage (72 h) EMT induced by METs and its attenuation by intervention.** (H-J)** Representative immunofluorescence images of EMT-related proteins **(H)** E-cadherin, **(I)** N-cadherin, and **(J)** Fibronectin in epithelial cells treated for 72 h under the same conditions (scale bars, 50 μm). **(K-L)** Representative Western blots and corresponding quantification of EMT-related (E-cadherin, N-cadherin, Vimentin) and fibrosis-related (Collagen I, α-SMA) proteins under the same treatment conditions. **(M-O)** Quantification of fluorescence intensity for E-cadherin, N-cadherin, and Fibronectin. Data are presented as mean ± SD. Statistical significance is indicated as ns, not significant; *P < 0.05, **P < 0.01, ***P < 0.001.

Using this system, macrophages were stimulated with recombinant CXCL8 to induce pronounced METosis, and the resulting conditioned medium (CM-METs) was collected for treatment of lung epithelial cells (Figure 8A). First, we assessed the effect of CM-METs on epithelial barrier integrity. Immunofluorescence analysis revealed that exposure to CM-METs disrupted the membrane localization of the tight junction proteins zonula occludens-1 (ZO-1) and Occludin. Importantly, this barrier disruption was significantly attenuated either by pretreatment with a CXCR2 inhibitor to block METs formation or by treatment with DNase I to degrade preformed METs structures (Figure 8, B-E). Western blot analysis corroborated these findings, demonstrating a reduction of ZO-1 and Occludin protein levels, which was ameliorated by METosis intervention (Figure 8, F and G).

Given the marked EMT features and fibrotic remodeling observed in the late stage of the combined lung injury model *in vivo*, we next investigated whether METs-containing conditioned medium could induce EMT-like changes, a key process in fibrosis. Immunofluorescence analysis revealed that epithelial cells treated with CM-METs for 72 h displayed EMT-like phenotypic changes, including decreased E-cadherin expression and increased N-cadherin and fibronectin signals (Figure 8, H-J and M-O). These changes were attenuated either by blocking METs formation with a CXCR2 inhibitor or by degrading preformed MET structures with DNase I (Figure 8, H-J and M-O). Consistently, Western blot analysis showed that CM-METs reduced E-cadherin levels while increasing the expression of N-cadherin, Vimentin, Collagen I, and α-SMA, suggesting EMT- and fibrosis-related molecular changes (Figure 8, K and L). Notably, these alterations were also attenuated by inhibition of METs formation or degradation of preformed METs structures (Figure 8, K and L).

### Cordycepin Attenuates Combined Lung Injury in Association with Reduced Macrophage Extracellular Trap Formation

Our longitudinal characterization showed that combined lung injury follows a distinct biphasic pathological course, with an initial pronounced inflammatory response followed by progressive fibrosis. This specific trajectory suggests the need for a therapeutic intervention with dual anti-inflammatory and anti-fibrotic properties. Based on our group's previous findings that Cordycepin has radioprotective 33, anti-inflammatory 34, and anti-fibrotic potential 35, we selected it as a candidate drug to systematically evaluate its efficacy in this model.

**Figure 9:**
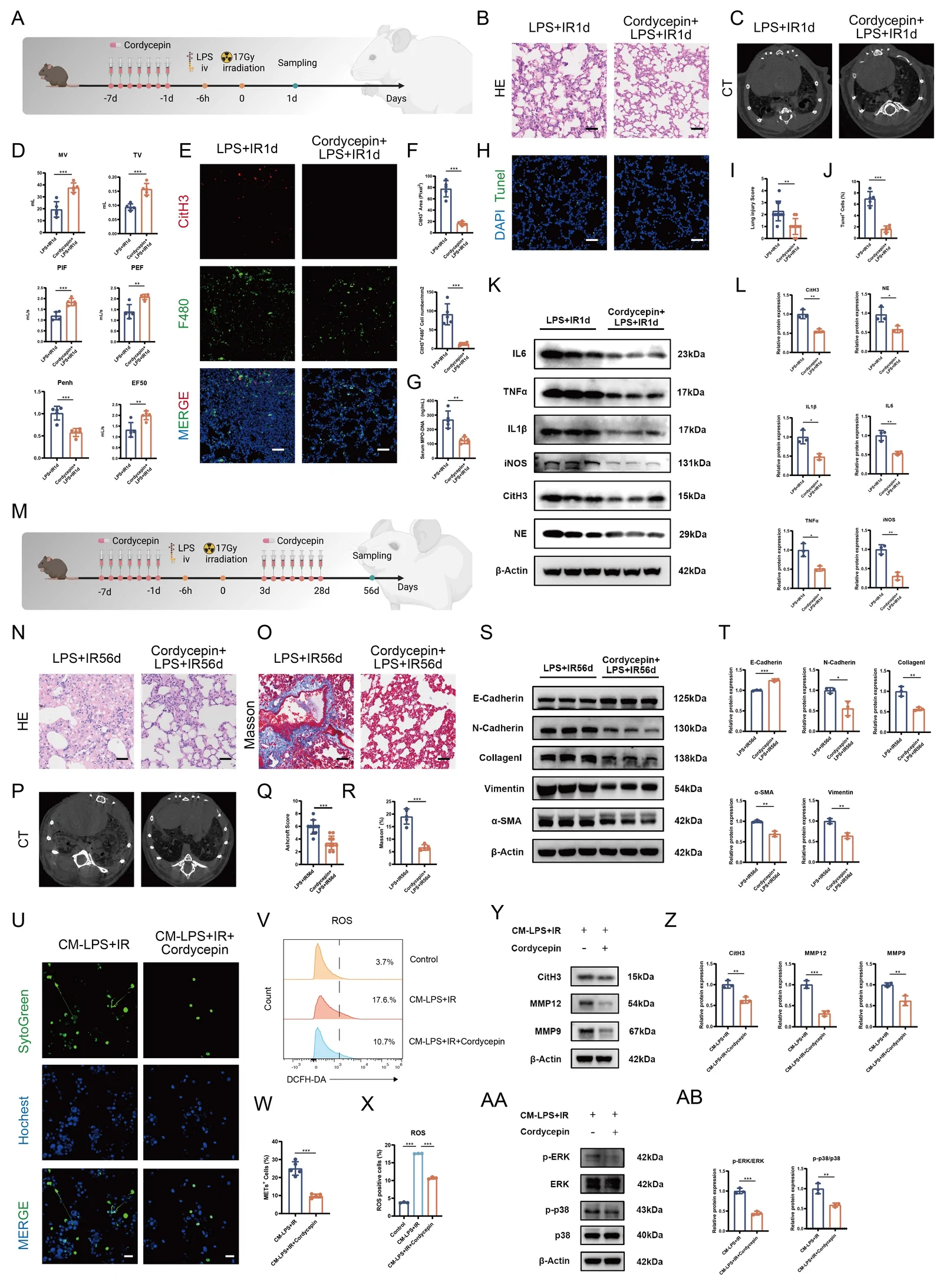
** Cordycepin attenuates combined lung injury in association with reduced METosis and p38/ERK activation. (A)** Schematic of the prophylactic treatment regimen with Cordycepin for the acute phase of combined lung injury. **(B-L)** Cordycepin alleviates the acute phase of combined lung injury at day 1 post-injury.** (B)** Representative H&E staining images (scale bars, 50 μm) and **(C)** Micro-CT scans of lung tissues. **(D)** Lung function assessment, including MV, TV, PIF, PEF, EF50, and Penh. **(E)** Representative immunofluorescence co-staining of F4/80 and CitH3 (scale bars, 50 μm). **(F)** Quantification of CitH3 fluorescence intensity and F4/80⁺CitH3⁺ double-positive cells. **(G)** Quantification of serum MPO-DNA levels. **(H)** Representative images of TUNEL staining (scale bars, 50 μm).** (I)** Quantification of lung injury scores based on H&E staining. **(J)** Quantification of TUNEL-positive cells.** (K-L)** Representative Western blots and corresponding quantification of METosis-related and pro-inflammatory proteins at day 1. **(M)** Schematic of the prophylactic and therapeutic Cordycepin regimen targeting late-stage fibrosis in the combined lung injury model. **(N-T)** Cordycepin attenuates late-stage pulmonary fibrosis at day 56 post-injury. Representative **(N)** H&E staining, **(O)** Masson's trichrome staining, and **(P)** Micro-CT images of lung tissues at day 56 (H&E and Masson's trichrome scale bars, 50 μm). **(Q-R)** Quantification of **(Q)** Ashcroft fibrosis scores and **(R)** Masson-positive area. **(S-T)** Representative Western blots and corresponding quantification of EMT-related proteins (E-cadherin, N-cadherin, Vimentin) and fibrosis-related proteins (Collagen I, α-SMA). **(U-AB)** Cordycepin attenuates CM-LPS+IR-induced METosis and ROS production in macrophages and reduces p38/ERK pathway activation. **(U)** Representative immunofluorescence images of METs formation (SytoGreen/Hoechst staining) in macrophages stimulated with CM from LPS+IR-treated endothelial cells, with or without Cordycepin treatment (scale bars, 100 μm). **(V)** Representative flow cytometry plots of ROS production in macrophages under the same conditions. **(W-X)** Quantification of **(W)** MET-positive cells and **(X)** ROS-positive cells from (U) and (V), respectively. **(Y-Z)** Representative Western blots and corresponding quantification of METosis-related proteins (CitH3, MMP12, and MMP9) in macrophages under the same conditions. **(AA-AB)** Representative Western blots and corresponding quantification of p-p38/p38 and p-ERK/ERK in macrophages under the same conditions. Data are presented as mean ± SD. Statistical significance is indicated as ns, not significant; *P < 0.05, **P < 0.01, ***P < 0.001.

We first assessed the protective efficacy of Cordycepin in the acute injury phase. Following the regimen established in our previous work, mice received prophylactic Cordycepin treatment before model establishment (Figure 9A). Both histopathological and CT assessments confirmed that Cordycepin treatment markedly attenuated the pulmonary interstitial edema and inflammatory cell infiltration (Figure 9, B-C and I). At the cellular level, TUNEL staining showed a reduction in cell death after treatment (Figure 9, H and J). Consistently, lung function tests indicated that Cordycepin treatment ameliorated respiratory dysfunction, as evidenced by improved respiratory parameters (Figure 9D). Mechanistically, Cordycepin suppressed METosis, an important pathological process, as shown by reduced CitH3 fluorescence intensity and a reduction in CitH3⁺F4/80⁺ populations (Figure 9, E and F). Consistently, Cordycepin also reduced serum levels of MPO-DNA complexes, suggesting reduced systemic extracellular trap release (Figure 9G). Western blot analysis further confirmed that the expression of both pro-inflammatory proteins (IL-6, TNF-α, IL-1β, iNOS) and METosis-related proteins (CitH3, NE) was reduced after treatment (Figure 9, K and L). In addition, we further evaluated a single-dose therapeutic regimen in which Cordycepin was administered at the time of model establishment. Histopathological and CT assessments consistently demonstrated that this regimen alleviated lung pathological injury (Supplemental Figure 17, A and B), suggesting that Cordycepin also confers protective effects when administered during the acute injury phase, in addition to its prophylactic benefit.

Next, we evaluated the therapeutic potential of Cordycepin against late-stage pulmonary fibrosis (Figure 9M). Evaluation at day 56 revealed that this intervention exerted anti-fibrotic effects. Histopathological and CT assessments demonstrated a significant attenuation of fibrotic pathology in the treatment group. Specifically, histopathological analysis revealed reduced architectural distortion, while CT scans correspondingly displayed a decrease in high-density opacities and reticular patterns (Figure 9, N and P-Q). Masson's trichrome staining also confirmed a significant reduction in collagen deposition (Figure 9, O and R). At the molecular level, Western blot analysis showed that Cordycepin attenuated EMT- and fibrosis-related protein expression changes, as indicated by the upregulation of E-cadherin and downregulation of N-cadherin and Vimentin. Concurrently, the expression of fibrotic proteins α-SMA and Collagen I was also reduced (Figure 9, S and T).

To further explore the mechanism underlying the effects of Cordycepin, we performed *in vitro* mechanistic studies. The results suggested that Cordycepin attenuated METosis and intracellular ROS production in macrophages exposed to conditioned medium from LPS+IR-treated endothelial cells (CM-LPS+IR) (Figure 9, U-X). Western blot analysis further showed that Cordycepin reduced the expression of METosis-related proteins (CitH3, MMP12, MMP9) (Figure 9, Y and Z).

As Cordycepin was found to suppress METosis *in vivo*, and our previous results indicated that the CXCR2-p38/ERK pathway is involved in CM-LPS+IR-induced METosis, we next investigated whether Cordycepin modulates this signaling axis. Molecular docking analysis suggested potential interactions between Cordycepin and p38, ERK1, and ERK2 (Supplemental Figure 18, A-C). Consistent with this, Western blot analysis showed that Cordycepin reduced the phosphorylation levels of both p38 and ERK in macrophages stimulated with CM-LPS+IR (Figure 9, AA and AB). A similar decrease in p-p38/p38 and p-ERK/ERK was also observed in lung tissues after Cordycepin treatment (Supplemental Figure 18, D-E), further supporting the involvement of p38/ERK pathway modulation in its protective effects *in vivo*.

These results collectively suggest that Cordycepin exerts its protective effects by suppressing p38/ERK pathway activation, thereby attenuating METosis and alleviating both acute inflammatory injury and subsequent fibrotic progression.

## Discussion

**Figure 10:**
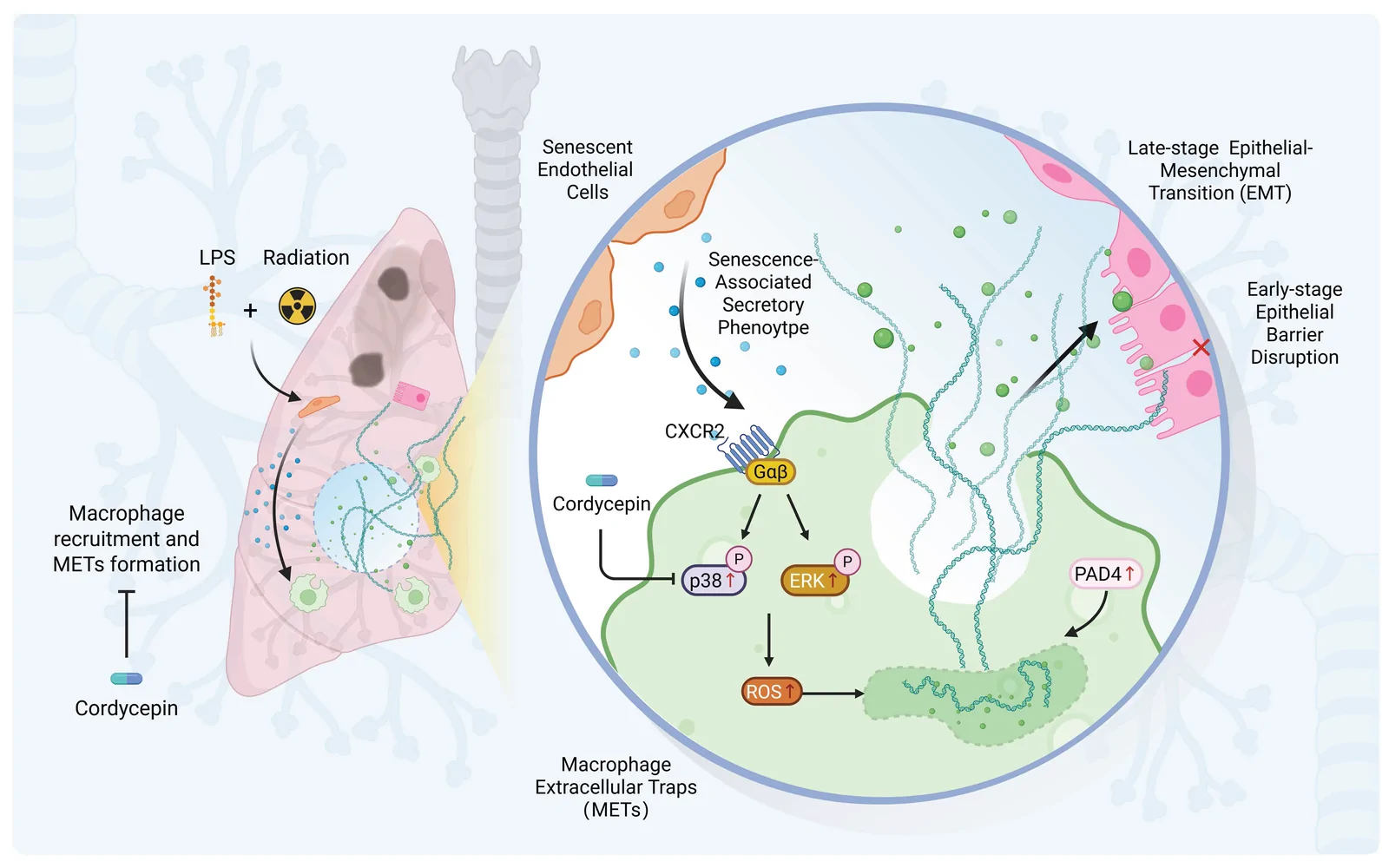
** Schematic illustration of the proposed endothelial senescence-associated secretory signaling-METosis axis in combined lung injury.** The combination of LPS and thoracic irradiation induces an early endothelial senescence-associated phenotype and a CXC chemokine-enriched secretory microenvironment. These secretory signals engage CXCR2 on macrophages and are associated with activation of the p38/ERK signaling pathway and increased ROS production, thereby promoting METosis. METs may contribute to early-stage epithelial barrier disruption and EMT-like epithelial remodeling, potentially linking acute lung injury to subsequent fibrotic remodeling. Cordycepin attenuates this pathological process by suppressing p38/ERK pathway activation, attenuating METosis, and alleviating combined lung injury.

The synergistic interplay between pre-existing inflammation and radiation injury represents a critical knowledge gap in understanding severe RILI. While clinical observations have long linked inflammatory comorbidities to poor radiotherapy outcomes, the precise molecular drivers remain elusive. In this study, we sought to address this gap by characterizing a potential endothelial senescence-associated secretory signaling-METosis axis. We demonstrate that METosis contributes to the synergistic pathology, which is mechanistically associated with senescence-associated endothelial cells releasing CXC chemokine-enriched secretory signals that activate the downstream CXCR2-p38/ERK-ROS signaling pathway. Furthermore, we identified METs as a potential mechanistic link between acute barrier disruption and late-stage fibrosis through the promotion of EMT-like epithelial remodeling. Finally, we showed that the natural bioactive compound Cordycepin exerts protective effects against both acute inflammation and chronic fibrosis in association with reduced p38/ERK pathway activation and attenuated METosis, thus providing experimental support for its further evaluation in high-risk patients. A schematic summary of the proposed mechanism is shown in Figure 10.

The establishment of our combined lung injury mouse model provides a framework for dissecting the complex interplay between pre-existing inflammation and radiation-induced lung injury. Notably, the biological consequences of this interplay appear to be highly context-dependent. Indeed, prior research has paradoxically identified LPS-mediated TLR4 signaling as a radioprotective mechanism under specific conditions. For instance, Zaidi *et al*. reported that post-irradiation treatment with LPS (50 μg/animal) attenuated lung injury, evidenced by reduced inflammatory cell infiltration and cytokine levels 9. Similarly, Guo *et al*. demonstrated that Monophosphoryl lipid A (MPLA), a detoxified derivative of LPS, conferred protection against irradiation by mitigating cellular apoptosis and DNA damage 36. Conversely, when inflammation manifests as a comorbidity outside this window of tolerance, it transforms into a potent radiosensitizer. For instance, Kalm *et al*. revealed that LPS pretreatment (0.3 mg/kg) sensitized juvenile brains to radiation, significantly exacerbating radiation-induced inflammation and cerebral injury 37. Similarly, Li *et al*. observed that prior irradiation aggravated LPS-induced pneumonia challenged 28 days later, a synergistic toxicity driven by the activation of the NLRP3 inflammasome pathway 38. However, a model specifically recapitulating pre-existing systemic inflammation prior to thoracic irradiation, which mirrors the prevalent high-risk clinical scenario, has been lacking. To address this, we established a murine model in which 2 mg/kg LPS was administered 6 h prior to 17 Gy irradiation, recapitulating the clinically observed synergistic pathology characterized by exacerbated acute pneumonitis and accelerated pulmonary fibrosis.

A distinctive and unexpected feature emerging from our study is the identification of METosis, rather than NETosis, as a prominent pathogenic process in the synergistic pathology of combined lung injury, a phenomenon that is rarely observed under either single injury alone. Similar to NETosis, METosis is typically associated with ROS generation and PAD4 enzyme activation 29. For a long time, research on ETosis has predominantly centered on NETosis, and the pathological role of NETs has been reported in various models of ALI 13,14 and pulmonary fibrosis 15,16. Research on METs in lung injury is still in its infancy, and our study provides evidence supporting the involvement of METs in this specific pathological context of acute lung injury. More importantly, our findings further support the involvement of METosis in disease progression. By suppressing METosis through pharmacological and genetic targeting of PAD4, we observed attenuation of combined lung injury. Our further investigation revealed that METs may function as potential dual-phase mediators, contributing to both acute tissue injury and chronic remodeling. In the acute phase, METs act as important mediators of epithelial barrier disruption by disrupting the tight junction proteins ZO-1 and Occludin, an effect that can be attenuated by inhibition of METs formation or degradation of METs structures. This observation offers a possible molecular explanation for the marked alveolar edema observed in early combined injury. In addition, the ability of METs to promote **E**MT-like epithelial remodeling suggests that they may serve as a mechanistic link between the acute inflammatory phase and the late fibrotic phase of the disease. These findings suggest that METs may not merely mediate transient damage, but may also contribute to epithelial remodeling associated with subsequent fibrosis, highlighting their potential role in both the acute and chronic phases of lung injury.

Tracing the upstream regulatory signals of this pathology, we identified an early endothelial senescence-associated phenotype as a potential upstream event associated with METosis. Distinct from replicative senescence, an age-associated and largely irreversible cell-cycle arrest, stress-induced senescence is triggered by diverse stress stimuli, including oxidative stress, DNA damage, and pro-inflammatory signals 39. Unlike the classical paradigm that frames senescence as a protracted and stable state 40, our findings suggest that the combination of LPS and irradiation rapidly induces an endothelial senescence-associated program within 24 h, which represents an early feature of the combined injury. This stress-induced senescence-associated state was accompanied by the emergence of a SASP enriched in pro-inflammatory CXC chemokines, including CXCL1, CXCL2, CXCL3, and CXCL8. Thus, the impact of this state may extend beyond cell-cycle arrest to modulation of the local immune microenvironment. While transient SASP signaling can be beneficial by orchestrating immune recruitment and tissue repair, excessive or dysregulated SASP output is increasingly recognized as maladaptive, amplifying inflammation, perturbing immune homeostasis, and promoting fibro-inflammatory remodeling 41. Notably, Binet *et al*. provided seminal evidence that senescent vasculature releases SASP signals associated with NETosis induction 27. Consistent with this emerging concept, our study suggests that the endothelial senescence-associated secretory milieu may provide upstream-associated stimulus for METosis, with its CXC chemokine-rich secretome promoting macrophage recruitment, polarization, and METosis.

Having identified a CXC chemokine-enriched endothelial senescence-associated secretory profile as a potential upstream signal associated with METosis, we further examined the signaling events involved in this process. Our findings suggest that the pro-METosis activity of the endothelial secretome is mediated largely by CXC chemokines acting through CXCR2, as neutralization of CXCL8 or CXCL1/2/3, as well as genetic or pharmacological inhibition of CXCR2, consistently attenuated METosis. We further found that ROS generation contributes to this response, since NADPH oxidase inhibition reduced both intracellular ROS levels and METosis. In addition, pharmacological inhibition experiments indicated that p38 and ERK, but not PI3K, are involved in the downstream signaling events potentially linking CXCR2 activation to ROS production and METosis. Together, these findings broaden our understanding of METosis regulation and support a mechanistic link between endothelial senescence-associated secretory signaling and the oxidative response involved in METosis.

The characterization of this potential pathogenic mechanism provides a theoretical basis for the development of potential therapeutic strategies. Our combined injury model exhibits a distinct biphasic course characterized by early inflammation followed by late fibrosis, indicating that effective intervention should ideally possess both anti-inflammatory and anti-fibrotic properties. Based on our group's previous findings, we selected Cordycepin 33 as a candidate drug for validation. Our results showed that Cordycepin not only attenuated lung injury and inflammation in the acute phase but also reduced the development of pulmonary fibrosis in the chronic phase. More importantly, our *in vivo* and *in vitro* data suggest that Cordycepin exerts its protective effects by suppressing p38/ERK pathway activation, thereby attenuating METosis, an important pathological process. Given the current lack of effective pharmacological interventions specifically targeting METosis, our findings not only reveal a potential therapeutic benefit of Cordycepin in this combined lung injury model, but also support its further evaluation as a candidate compound for modulating METosis-associated injury. Collectively, these results provide supporting evidence for the therapeutic potential of targeting METosis and offer a potential translational rationale for developing METosis-targeted interventions in high-risk populations.

Despite these findings, several limitations should be noted. First, although our data indicate that CitH3⁺F4/80⁺ macrophages are the predominant extracellular trap-forming population in this model, the PAD4-targeting approaches used in this study have limited cell-type specificity. Both Cl-amidine treatment and global PAD4 knockout broadly suppress PAD4-dependent extracellular trap formation, and a minor contribution from other PAD4-dependent processes, including residual NETosis, remains possible. Future studies using macrophage-lineage-specific PAD4 knockout mice, such as Lyz2-Cre; PAD4^fl/fl^ mice, would help further define the macrophage-specific role of PAD4-dependent METosis in combined lung injury. Second, although our data show that the combination of LPS and irradiation induces an early endothelial senescence-associated phenotype and that the resulting endothelial secretome promotes macrophage METosis, we did not directly eliminate senescent endothelial cells using senolytics or genetic clearance of p16-positive cells. Therefore, whether endothelial senescence is causally required for METosis and lung injury remains to be established. Third, our analysis of METosis was mainly focused on the early acute phase, and continuous multi-time-point tracking of MET formation and resolution remains to be further explored. Such temporal analyses would help clarify how METosis evolves during the transition from acute inflammation to chronic fibrotic remodeling. In addition, we did not directly test whether delayed degradation of METs or stage-specific inhibition of METosis could attenuate late-stage pulmonary fibrosis. Therefore, the direct link between early METosis and late fibrotic remodeling remains to be established. Fourth, although MET-containing macrophage conditioned medium disrupted epithelial barrier integrity and induced EMT-like changes, the precise MET-associated effector components responsible for these effects remain to be identified, leaving the molecular link between METs and epithelial remodeling incompletely defined. Fifth, although our data suggest that endothelial secretome-induced METosis is largely associated with CXC chemokine-CXCR2 signaling, we cannot completely exclude the potential contribution of other SASP-related factors, such as IL6 and IL1β, to the induction of METosis. Sixth, although Cordycepin showed protective effects in both acute and chronic injury settings and was associated with reduced p38/ERK activation, we did not perform direct drug-protein interaction experiments to determine whether Cordycepin directly binds to p38, ERK1, or ERK2, and other mechanisms cannot be excluded. Seventh, several pharmacological inhibitors used in this study may have off-target effects. Although these inhibitors were useful as pathway-probing tools, we did not perform genetic knockout experiments or *in vivo* pharmacological interventions targeting CXCR2, p38, ERK, or NADPH oxidase. Therefore, the pathway-specific contribution of CXCR2-p38/ERK-ROS signaling to METosis and combined lung injury *in vivo* remains to be further validated. Finally, although Cordycepin showed protective effects in both acute and chronic injury settings, the treatment regimens used in this study primarily evaluated prophylactic and acute-phase intervention strategies. Delayed post-injury therapeutic administration, which would more closely resemble clinical intervention after disease onset, warrants further investigation. Moreover, validation using clinical samples from patients with radiation-induced lung injury or pre-existing inflammatory conditions would further strengthen the translational relevance of our findings. Future studies incorporating patient-derived specimens may help clarify the clinical relevance of the endothelial senescence-associated secretory signaling-METosis axis and the translational potential of targeting this pathway.

In summary, our work establishes a clinically relevant combined-injury model and supports a potential endothelial senescence-associated secretory signaling-METosis axis in severe RILI, in which a CXC chemokine-enriched secretory profile associated with endothelial senescence engages macrophage CXCR2 and activates the p38/ERK-ROS axis, thereby promoting METosis. By positioning METs as potential dual-phase mediators that link early epithelial barrier disruption to later EMT-like fibrotic remodeling, we highlight METosis as a candidate therapeutic target connecting acute inflammation with chronic tissue remodeling. Moreover, the efficacy of Cordycepin supports the concept that modulation of p38/ERK pathway activation may contribute to concurrent anti-inflammatory and anti-fibrotic effects by attenuating METosis. Together, these findings broaden our understanding of radiation lung injury and provide a potential translational framework for the development of METosis-targeted interventions in high-risk populations.

## Supplementary Material

Supplementary figures and tables.

## Data Availability

The sequencing data generated in this study have been deposited in the CNCB public database under BioProject accession number PRJCA062910.

## Abbreviations

ALI: acute lung injury
BALF: bronchoalveolar lavage fluid
CM: conditioned medium
DEGs: differentially expressed genes
EMT: epithelial-mesenchymal transition
ETs: extracellular traps
ETosis: extracellular traps formation
GO: Gene Ontology
GSEA: gene set enrichment analysis
IR: irradiation
KEGG: Kyoto Encyclopedia of Genes and Genomes
LPS: lipopolysaccharide
METs: macrophage extracellular traps
METosis: macrophage extracellular traps formation
NETs: neutrophil extracellular traps
PF: pulmonary fibrosis
RILI: radiation-induced lung injury
ROS: reactive oxygen species
RP: radiation pneumonitis
SASP: senescence-associated secretory phenotype
