# Supplementary material for: Endothelial Senescence-Associated Secretory Signaling Promotes Macrophage Extracellular Traps Formation and Contributes to the Exacerbation of Combined Lung Injury
Source: Int J Biol Sci. 2026 Jul 20;22(12):6790–813. doi: 10.7150/ijbs.133943 (PMC13412428; doi:10.7150/ijbs.133943)
Supplement: Supplementary file 1 — Supplementary figures and tables. [file ijbsv22p6790s1.pdf]

Supplementary Information

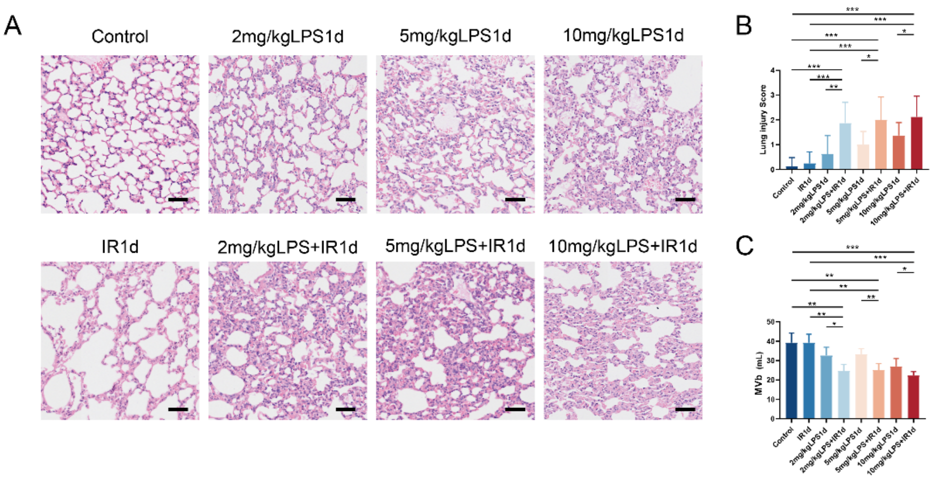

Supplemental Figure 1. Optimization of LPS dosage for the establishment of the combined lung injury model. (A) Representative H&E staining images of lungs at day 1. **(B)** Quantification of lung injury scores. **(C)** Quantification of lung function (MV).

A

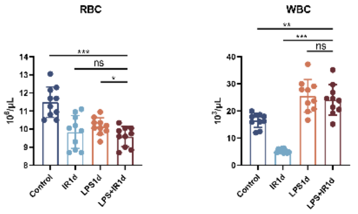

Supplemental Figure 2. Assessment of peripheral blood cell counts in combined lung injury at day 1. (A) Quantification of white blood cell (WBC) and red blood cell (RBC) counts in each group at day 1.

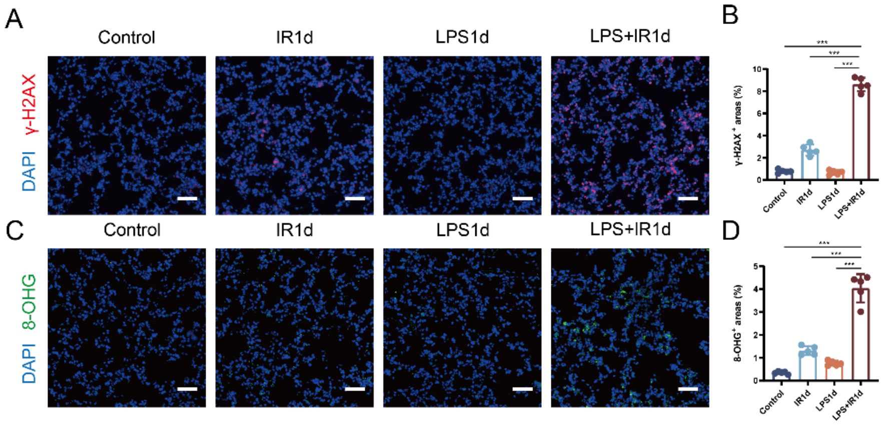

Supplemental Figure 3. Assessment of oxidative stress changes in combined lung injury at day 1. (A-B) Representative immunofluorescence images and quantification of γ-H2AX staining from the Control, IR, LPS, and LPS+IR groups at day 1. **(C-D)** Representative immunofluorescence images and quantification of 8-OHdG staining from the Control, IR, LPS, and LPS+IR groups at day 1.

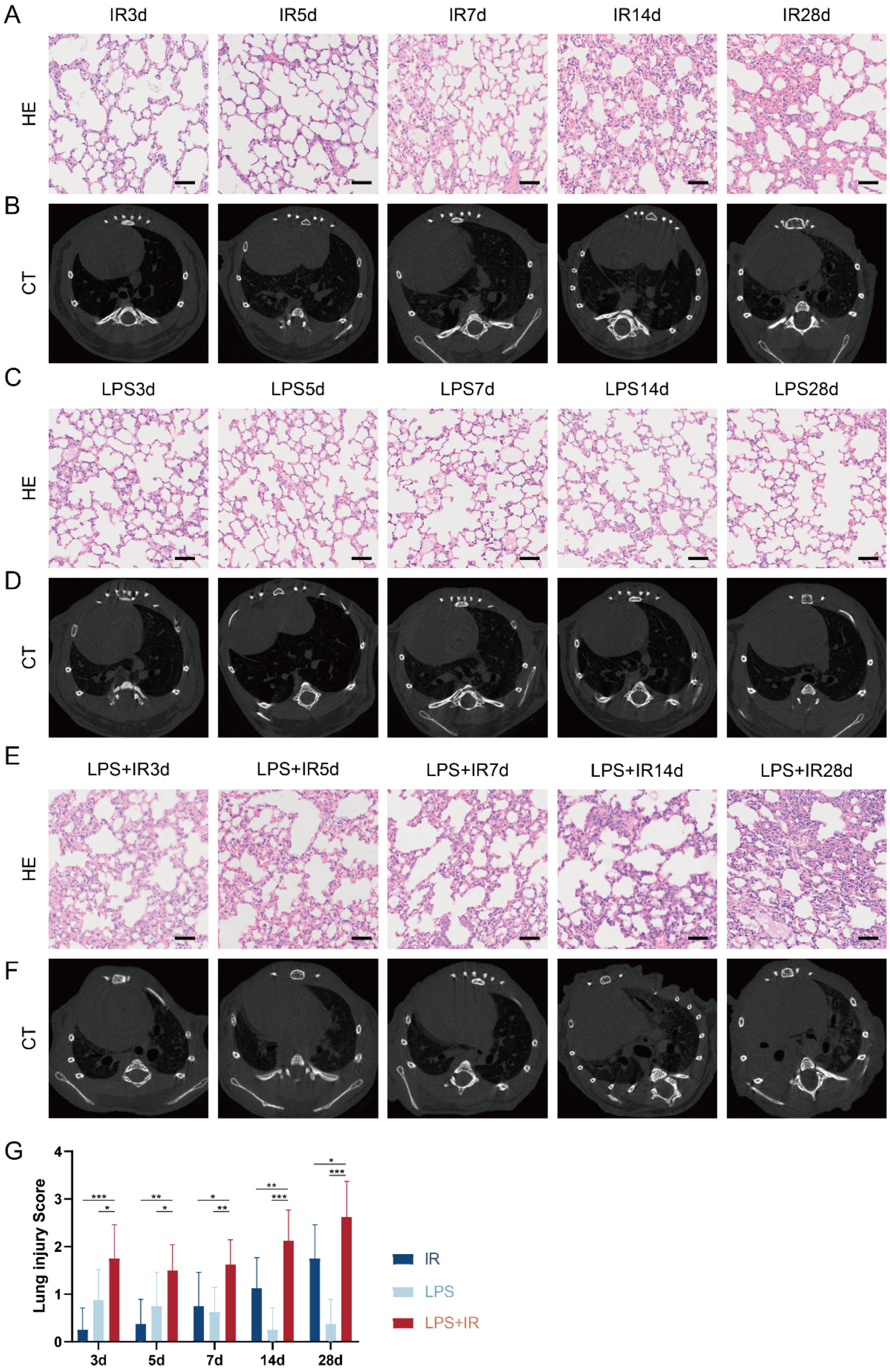

Supplemental Figure 4. Dynamic pathological evaluation at different time points. (A-B) Representative H&E staining and Micro-CT images of lungs from the IR group at days 3, 5, 7, 14, and 28. **(C-D)** Representative H&E staining and Micro-CT images

of lungs from the LPS group at the same time points. **(E-F)** Representative H&E staining and Micro-CT images of lungs from the LPS+IR group at the same time points. **(G)** Dynamic changes in lung injury scores for all groups over time.

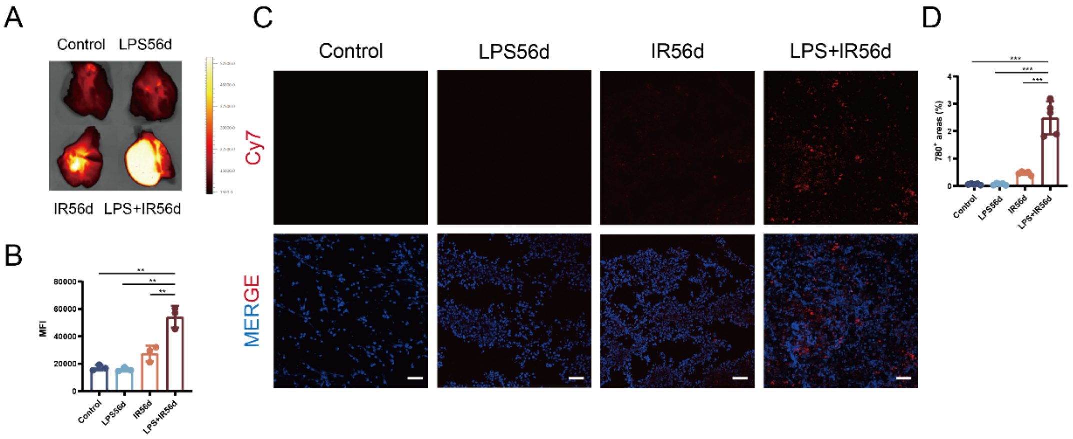

Supplemental Figure 5. Assessment of fibrosis in combined lung injury using the tracer IR780. **(A)** Representative ex vivo near-infrared images of lungs from Control, IR, LPS, and LPS+IR groups at day 56. **(B)** Quantification of mean fluorescence intensity from the ex vivo lung. **(C)** Representative near-infrared images of lung cryosections from the same groups at day 56. **(D)** Quantification of IR780-positive area from the cryosections.

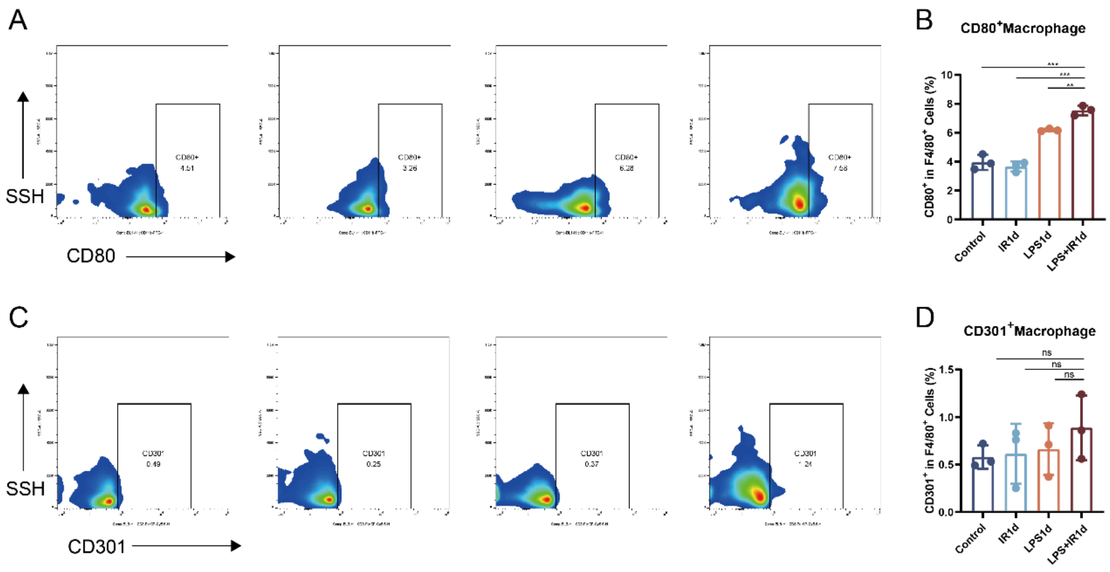

Supplemental Figure 6. Assessment of M1/M2 polarization of macrophages in combined lung injury at day 1. (A-B) Assessment of M1 polarization (CD80^{+}) in pulmonary macrophages by flow cytometry and its quantification. **(C-D)** Assessment of M2 polarization (CD301^{+}) in pulmonary macrophages by flow cytometry and its quantification.

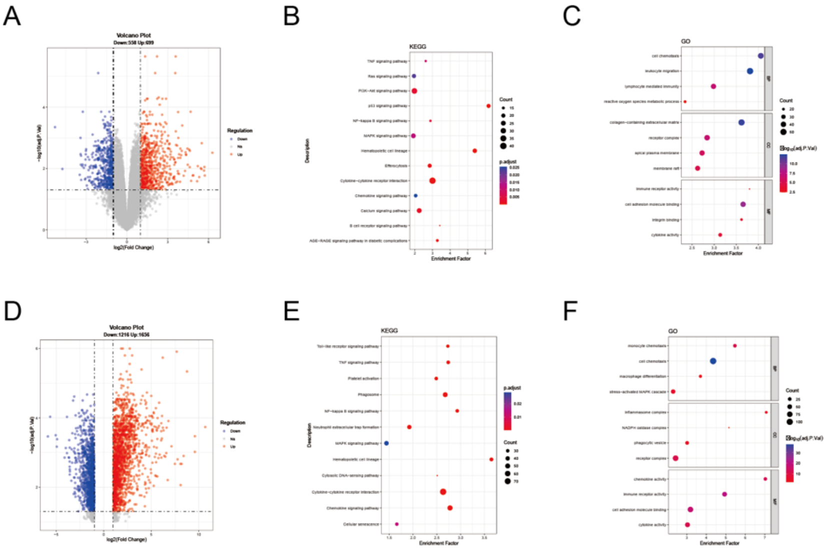

Supplemental Figure 7. Bioinformatic analysis of transcriptomes from single and combined injury groups. (A) Volcano plot of differentially expressed genes (DEGs) between the LPS+IR and LPS groups. **(B)** KEGG pathway and **(C)** GO functional enrichment analyses of these DEGs. **(D)** Volcano plot of DEGs between the LPS+IR and IR groups. **(E)** KEGG pathway and **(F)** GO functional enrichment analyses of these DEGs.

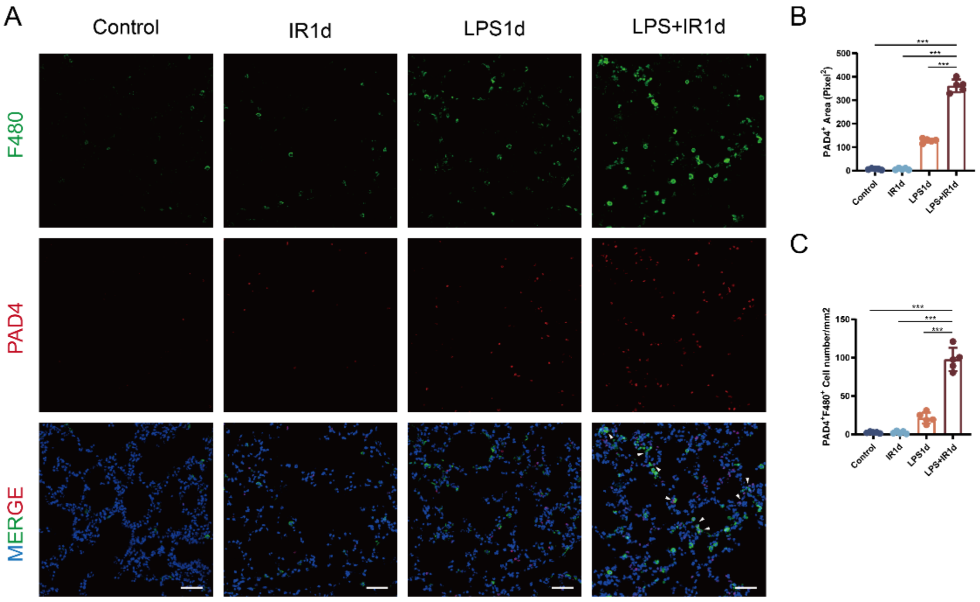

Supplemental Figure 8. Assessment of macrophage PAD4 expression in combined lung injury at day 1. (A) Representative immunofluorescence co-staining of PAD4 and F4/80 from the Control, IR, LPS, and LPS+IR groups at day 1. **(B)** Quantification of PAD4-positive area and **(C)** F4/80^{+} PAD4^{+} double-positive cells.

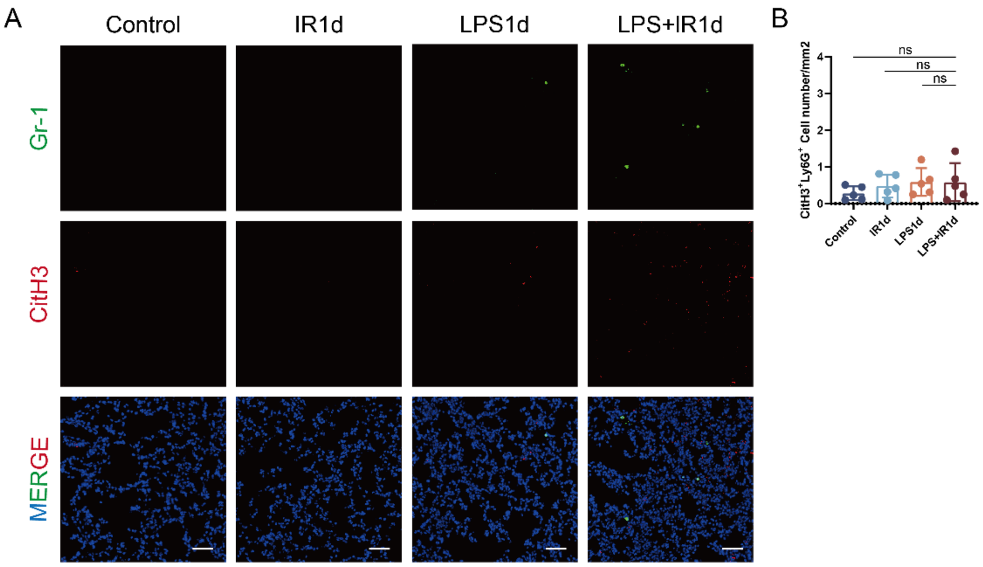

Supplemental Figure 9. Assessment of NETosis in combined lung injury at day 1. **(A)** Representative immunofluorescence co-staining of CitH3 and Ly6G from the Control, IR, LPS, and LPS+IR groups at day 1. **(B)** Quantification of CitH3^{+}Ly6G^{+} double-positive cells.

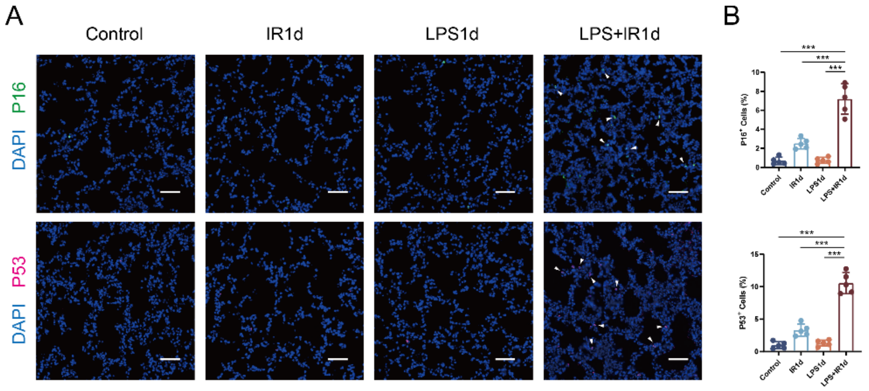

Supplemental Figure 10. **Assessment of cellular senescence in combined lung injury at day 1. (A-B)** Representative immunofluorescence images and quantification of cellular senescence markers P16 and P53 from the Control, IR, LPS, and LPS+IR groups at day 1.

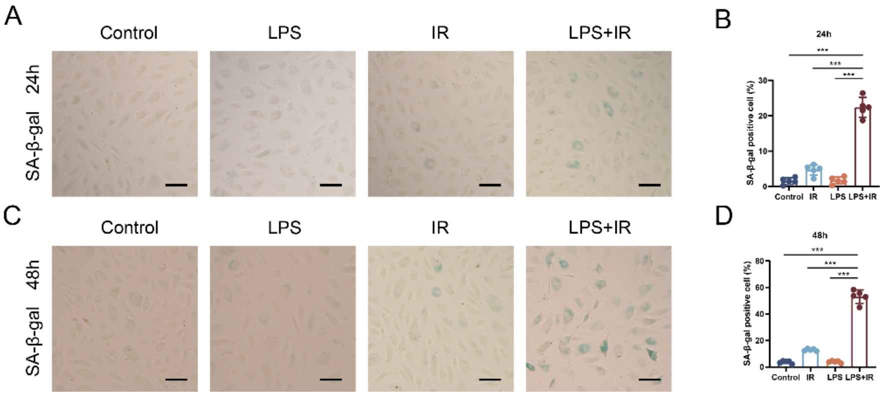

Supplemental Figure 11. SA-β-gal staining of endothelial cells in the Control, LPS, IR, and LPS+IR groups at 24 h and 48 h. (A) Representative SA-β-gal staining images of endothelial cells in the Control, LPS, IR, and LPS+IR groups at 24 h. **(B)** Quantification of the percentage of SA-β-gal-positive endothelial cells at 24 h. **(C)** Representative SA-β-gal staining images of endothelial cells in the Control, LPS, IR, and LPS+IR groups at 48 h. **(D)** Quantification of the percentage of SA-β-gal-positive endothelial cells at 48 h.

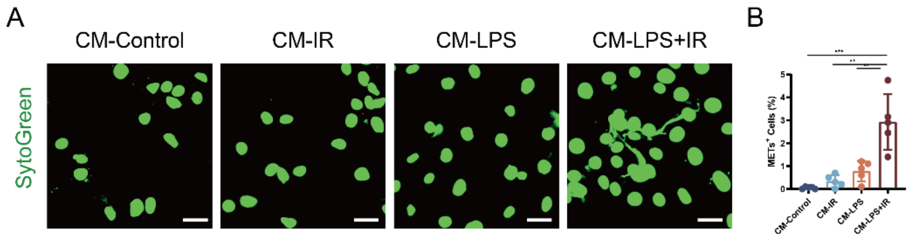

Supplemental Figure 12. Validation of endothelial secretome-induced METs formation using a primary murine co-culture system. (A) Representative immunofluorescence images show METs from primary murine bone marrow-derived macrophages (BMDMs) treated with conditioned medium (CM) from four groups of mouse pulmonary microvascular endothelial cells (MPVECs): untreated (Control), irradiated (IR), LPS-treated (LPS), or co-stimulated with LPS and IR (LPS+IR). **(B)** Quantification of METs-positive cells.

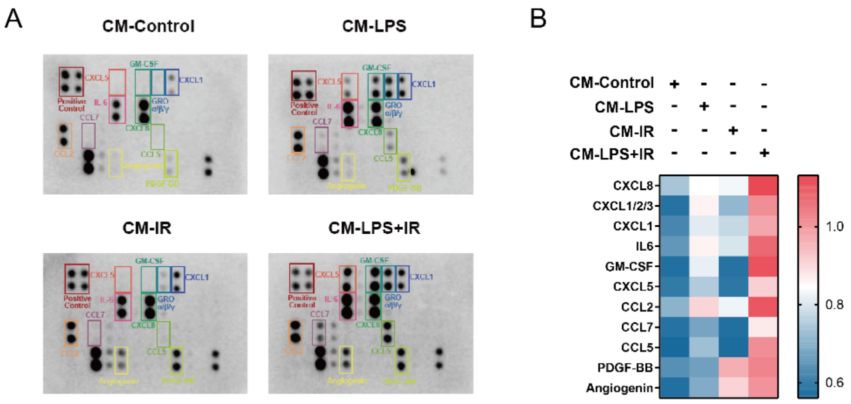

Supplemental Figure 13. Cytokine array analysis of endothelial cell-conditioned media at 24 h. (A) Representative cytokine array membranes of conditioned media collected from endothelial cells in the Control, LPS, IR, and LPS+IR groups at 24 h. **(B)** Heatmap showing the relative levels of differentially expressed secreted factors in conditioned media from each group.

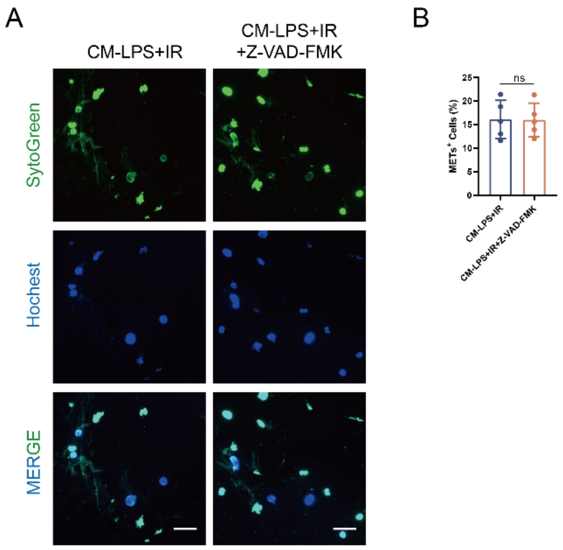

Supplemental Figure 14. Assessment of the effect of Z-VAD-FMK on CM-LPS+IR-induced METosis. (A) Representative immunofluorescence images of METs formation (SYTOX Green/Hoechst staining) in macrophages treated with CM-LPS+IR or CM-LPS+IR+Z-VAD-FMK. **(B)** Quantification of METs-positive cells from (A).

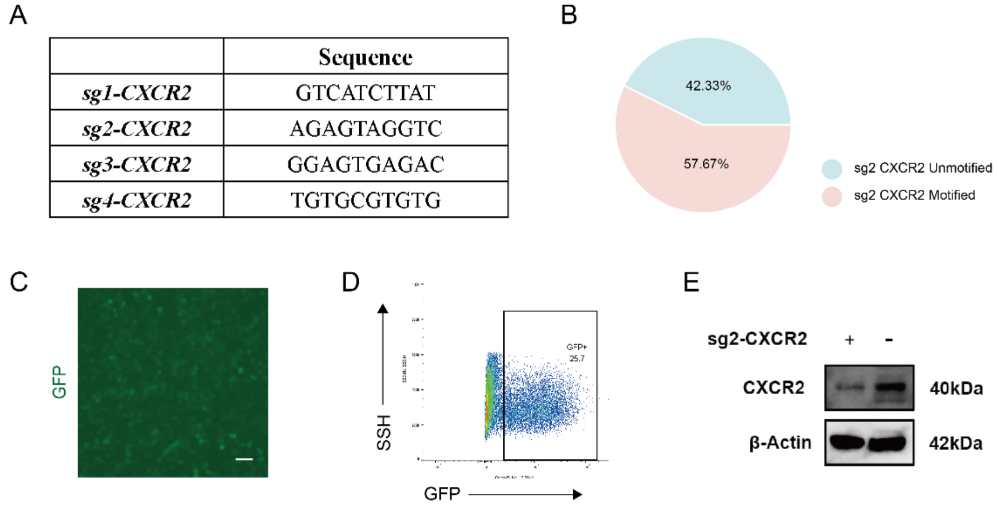

	Sequence
sg1-CXCR2	GTCATCTTAT
sg2-CXCR2	AGAGTAGGTC
sg3-CXCR2	GGAGTGAGAC
sg4-CXCR2	TGTGCGTGTG

Supplemental Figure 15. Generation and validation of CXCR2 knockdown macrophages. (A) Design of sgRNA sequences targeting CXCR2. **(B)** Evaluation of four sgRNA sequences post-transfection and selection of the most efficient one. **(C)** Assessment of transduction efficiency by immunofluorescence and **(D)** flow cytometry after lentiviral transduction. **(E)** Assessment of CXCR2 knockdown efficiency by Western blot after cell sorting.

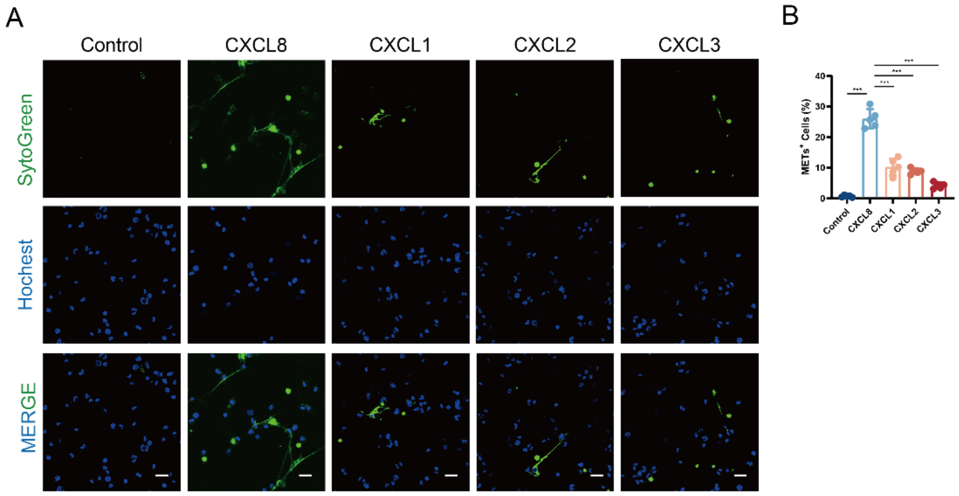

Supplemental Figure 16. CXCL8 induces pronounced METs formation among candidate SASP-related chemokines. (A) Representative immunofluorescence images of METs formation in macrophages stimulated with various recombinant chemokines (CXCL8, CXCL1, CXCL2, or CXCL3). **(B)** Quantification of the percentage of METs-positive cells.

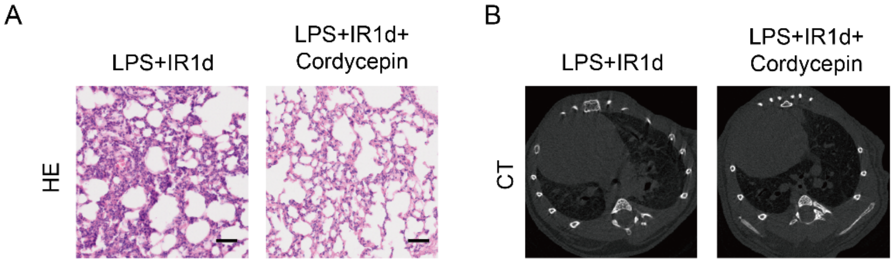

Supplemental Figure 17. Assessment of the therapeutic effect of single-dose Cordycepin treatment in combined lung injury at day 1. (A) Representative H&E staining images of lung tissues from the LPS+IR1d and Cordycepin+LPS+IR1d groups at day 1. **(B)** Representative micro-CT images of lung tissues from the same groups at day 1.

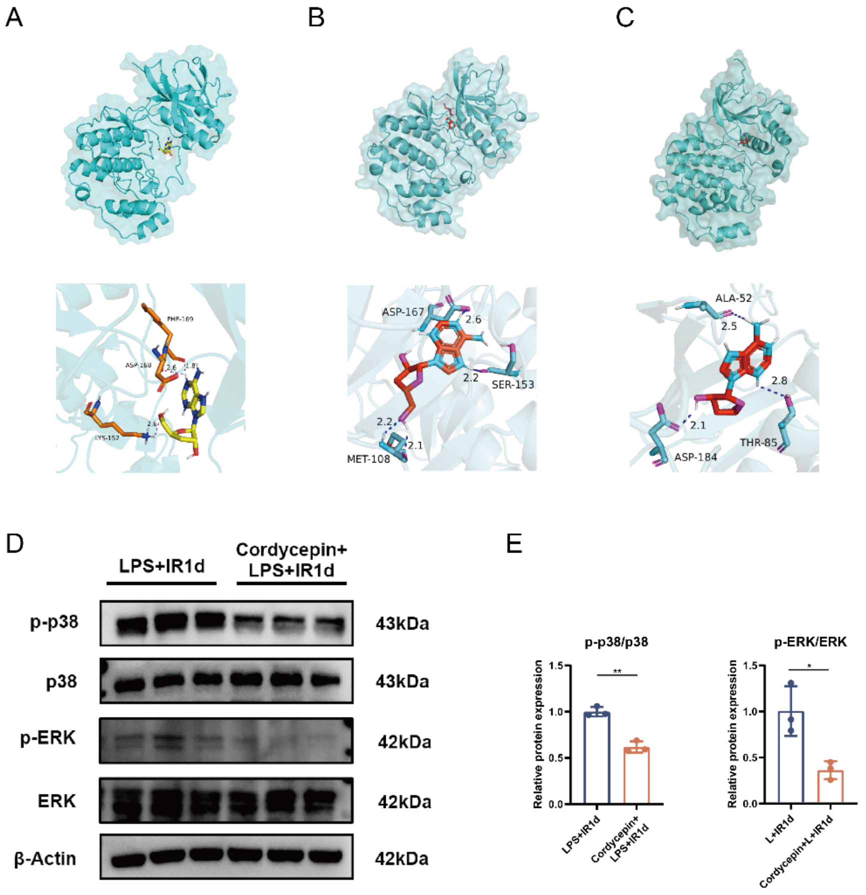

Supplemental Figure 18. Molecular docking analysis and assessment of p38/ERK pathway modulation by Cordycepin in vivo. (A-C) Molecular docking models showing the predicted interactions of Cordycepin with **(A)** p38, **(B)** ERK1, and **(C)** ERK2. **(D-E)** Representative Western blots and corresponding quantification of p-p38/p38 and p-ERK/ERK in lung tissues from the LPS+IR1d and Cordycepin+LPS+IR1d groups.

Table S1: Primer sequences used in PCR experiments

Gene	Species	Forward primer	Reverse primer
β-Actin	mouse	TGTACCCAGGCATTGCTGAC	AACGCAGCTCAGTAACAGTCC
IL-1β	mouse	GCAACTGTTCCTGAACTCAACT	ATCTTTTGGGGTCCGTCAACT
IL-6	mouse	GCTACCAAACTGGATATAATCAGGA	CCAGGTAGCTATGGTACTCCAGAA
TNF-α	mouse	CCCTCACACTCAGATCATCTTCT	GCTACGACGTGGGCTACAG
P16	mouse	CTTCGCCGAGCAGTTTCGT	TCAATCCCATCAGCCATTTCC
P21	mouse	CGAGAACGGTGGAACTTTGAC	CCAGGGCTCAGGTAGACCTT
P53	mouse	GAGGTTGGCTCTGACTGTACC	TCCGTCCCAGTAGATTACCAC
GAPDH	human	GGAGCGAGATCCCTCCAAAA	GCTGTTGTCATACTTCTCATGG
P16	human	CCCAACGCACCGAATAGTTA	ACCAGCGTGTCCAGGAAG
P21	human	CGATGGAACTTCGACTTTGTCA	GCACAAGGGTACAAGACAGTG
P53	human	GCGTAAACGCTTCGAGATGTT	TTTTTATGGCGGGAAGTAGACTG

Table S2: Antibodies used in WB experiments

Antibodies	Cat.number	Company	Dilution Ratio
IL-1β	12507S	CST	1:1000
TNF-α	11948S	CST	1:1000
IL-6	ab290735	abcam	1:1000
iNOS	A3774	Abclonal	1:1000
E-Cadherin	3195S	CST	1:1000
N-Cadherin	13116S	CST	1:1000
Collagen I	PA5-95137	Invitrogen	1:1000
Vimentin	ab92547	abcam	1:5000
CitH3	ab281584	abcam	1:1000
PADI4	17373-1-AP	proteintech	1:1000
Neutrophil Elastase	61928S	CST	1:1000
MMP12	22989-1-AP	proteintech	1:1000
MMP9	10375-2-AP	proteintech	1:1000
Phospho-p38 MAPK (Thr180/Tyr182)	9216S	CST	1:1000
p38 MAPK	8690S	CST	1:1000
Phospho-p44/42 MAPK (Erk1/2) (Thr202/Tyr204)	4370S	CST	1:1000
p44/42 MAPK (Erk1/2)	9107S	CST	1:1000
Phospho-Akt (Ser473)	4060S	CST	1:1000
Akt	9272S	CST	1:1000
ZO-1	21773-1-AP	proteintech	1:1000
Occludin	27260-1-AP	proteintech	1:1000
α-SMA	ab5694	abcam	1:2000
CXCR2	20634-1-AP	proteintech	1:1000

CXCL8/IL-8	27095-1-AP	proteintech	1:1000
CXCL1	12335-1-AP	proteintech	1:1000
CXCL2	26791-1-AP	proteintech	1:1000
P16	ab54210	abcam	1:1000
P21	ab188224	abcam	1:1000
P53	sc-126	Santa Cruz	1:1000
HRP-conjugated β-Actin	AC028	Abclonal	1:5000

Table S3: Antibodies used in IF experiments

Antibodies	Cat.number	Company	Dilution Ratio
α-SMA	ab5694	abcam	1:200
Gr-1	ab25377	abcam	1:200
F4/80	30325S	CST	1:200
iNOS	A3774	Abclonal	1:200
SFTPC	PA5-71680	Invitrogen	1:50
SCGB1A1	DF6581	Affinity	1:200
γ-H2AX	ab81299	abcam	1:200
8-OHdG	ab62623	abcam	1:200
CitH3	ab281584	abcam	1:200
PADI4	17373-1-AP	proteintech	1:200
MMP12	22989-1-AP	proteintech	1:200
E-Cadherin	20874-1-AP	proteintech	1:200
N-Cadherin	22018-1-AP	proteintech	1:200
ZO1	21773-1-AP	proteintech	1:200
Occludin	27260-1-AP	proteintech	1:200
Fibronectin	ab268020	abcam	1:200
P16	ab54210	abcam	1:200
P21	ab188224	abcam	1:200
P53	sc-126	santa cruz	1:50
CD31	ab28364	abcam	1:200
Goat Anti-Rabbit IgG H&L (Alexa Fluor555)	ab150078	abcam	1:800
Goat Anti-Rabbit IgG H&L (Alexa Fluor488)	ab150077	abcam	1:800
Goat Anti-Mouse IgG H&L (Alexa Fluor647)	ab150115	abcam	1:800
Goat Anti-Rat IgG H&L (Alexa Fluor488)	ab150165	abcam	1:800
Goat Anti-Rat IgG H&L (Alexa Fluor647)	A-21247	Invitrogen	1:800
SYTOX Green	S7020	Thermo Fisher	1:4000

Table S4: Antibodies used in FC experiments

**Antibodies**	**Cat.number**	**Company**	**Dilution Ratio**
PE/Cyanine7 anti-Nos2 (iNOS) Antibody	696813	Biolegend	1:200
FITC anti-mouse CD80 Antibody	104705	Biolegend	1:200
PerCP/Cyanine5.5 anti-mouse CD301	145709	Biolegend	1:200
PE Anti-mouse CD19	557399	BD Biosciences	1:200
APC Anti-mouse CD3e	553066	BD Biosciences	1:200
Brilliant Violet 421™ anti-mouse CD64 (FcγRI)	139332	Biolegend	1:200
APC anti-mouse CD45	103112	Biolegend	1:200
Brilliant Violet 421™ anti-mouse CD45	103134	Biolegend	1:200
FITC anti-mouse/human CD11b	101206	Biolegend	1:200
PE/Cyanine5 anti-mouse CD11c	117307	Biolegend	1:200
PE anti-mouse CD115 (CSF-1R)	135505	Biolegend	1:200
PE/Cyanine7 anti-mouse Ly-6G	127618	Biolegend	1:200
Brilliant Violet 421™ anti-mouse F4/80	123131	Biolegend	1:200
FITC anti-mouse F4/80	123108	Biolegend	1:200
APC anti-mouse F4/80	123116	Biolegend	1:200
PE anti-mouse CD86	105007	Biolegend	1:200
APC/Cy7 anti-mouse Ly-6C	128026	Biolegend	1:200
7-AAD	420404	Biolegend	1:500
Zombie Aqua™ Fixable Viability Kit	423101	Biolegend	1:500
Trustain Fcx plus anti-mouse CD16/32	156604	Biolegend	1:500
